# Using the IUCN Red List to map threats to terrestrial vertebrates at global scale

**DOI:** 10.1038/s41559-021-01542-9

**Published:** 2021-08-30

**Authors:** Michael B. J. Harfoot, Alison Johnston, Andrew Balmford, Neil D. Burgess, Stuart H. M. Butchart, Maria P. Dias, Carolina Hazin, Craig Hilton-Taylor, Michael Hoffmann, Nick J. B. Isaac, Lars L. Iversen, Charlotte L. Outhwaite, Piero Visconti, Jonas Geldmann

**Affiliations:** 1 UN Environment Programme World Conservation Monitoring Centre (UNEP-WCMC), Cambridge, UK; 2 Cornell Lab of Ornithology, Ithaca, NY USA; 3 Conservation Science Group, Department of Zoology, University of Cambridge, Cambridge, UK; 4 Center for Macroecology, Evolution and Climate, Globe Institute, University of Copenhagen, Copenhagen, Denmark; 5 BirdLife International, Cambridge, UK; 6 MARE—Marine and Environmental Sciences Center, ISPA—Instituto Universitário, Lisbon, Portugal; 7 IUCN, Cambridge, UK; 8 Conservation and Policy, Zoological Society of London, London, UK; 9 UK Centre for Ecology & Hydrology, Crowmarsh Gifford, UK; 10 Department of Environmental Science, Policy, and Management, University of California, Berkeley, Berkeley, CA USA; 11 Centre for Biodiversity and Environment Research, University College London, London, UK; 12 IIASA—International Institute for Applied Systems Analysis, Laxenburg, Austria

**Keywords:** Environmental impact, Conservation biology

## Abstract

The Anthropocene is characterized by unparalleled human impact on other species, potentially ushering in the sixth mass extinction. Yet mitigation efforts remain hampered by limited information on the spatial patterns and intensity of the threats driving global biodiversity loss. Here we use expert-derived information from the International Union for Conservation of Nature Red List on threats to 23,271 species, representing all terrestrial amphibians, birds and mammals, to generate global maps of the six major threats to these groups: agriculture, hunting and trapping, logging, pollution, invasive species, and climate change. Our results show that agriculture and logging are pervasive in the tropics and that hunting and trapping is the most geographically widespread threat to mammals and birds. Additionally, current representations of human pressure underestimate the overall pressure on biodiversity, due to the exclusion of threats such as hunting and climate change. Alarmingly, this is particularly the case in areas of the highest biodiversity importance.

## Abstract (web summary)

Red List information is used to generate global maps of the likelihood of impacts on terrestrial vertebrates exerted by agriculture, hunting and trapping, logging, pollution, invasive species and climate change.

## Main

The world has entered the Anthropocene, characterized by unparalleled human impact on the global environment^1,2^ and associated with devastating biodiversity losses^3,4^. Despite this, we still have limited information about the spatial patterns and intensity of the threats responsible for this crisis^5,6^. This is particularly true for pressures such as overexploitation, pollution and invasive species, for which no suitable remotely sensed proxies exist^7–9^. Additionally, for threats where remotely sensed data are available, these data measure physical processes such as habitat conversion or the expansion of infrastructure^10–12^. However, the specific impact on species and habitats does not necessarily scale with the intensity of these processes but is highly context specific^13^. As a result, existing representations of pressures may not adequately capture impacts on ecosystems and species, and lack the detail required to target conservation actions efficiently^14,15^. Better information on the spatial intensity of threats and how they affect species on the ground is critically important for improving conservation responses^5^.

The International Union for Conservation of Nature (IUCN) Red List of Threatened Species (hereafter ‘Red List’) is one of the richest and most authoritative data sources in conservation^16^ and is derived from tens of thousands of hours from expert volunteer contributors worldwide^17^. To date, over 100,000 species have been assessed against the IUCN Red List Categories and Criteria, including all amphibians, birds and mammals^18^. For assessed species, experts have evaluated the threats affecting individual species using a standardized method and classification scheme^19^. However, information on the spatial occurrence of threats affecting a given species within its distribution is not collected systematically and is limited to documenting whether the species is affected by a given threat anywhere in its range. Additionally, there are no comprehensive spatial summaries of these threats to species.

**Extended Data Fig. 1:**
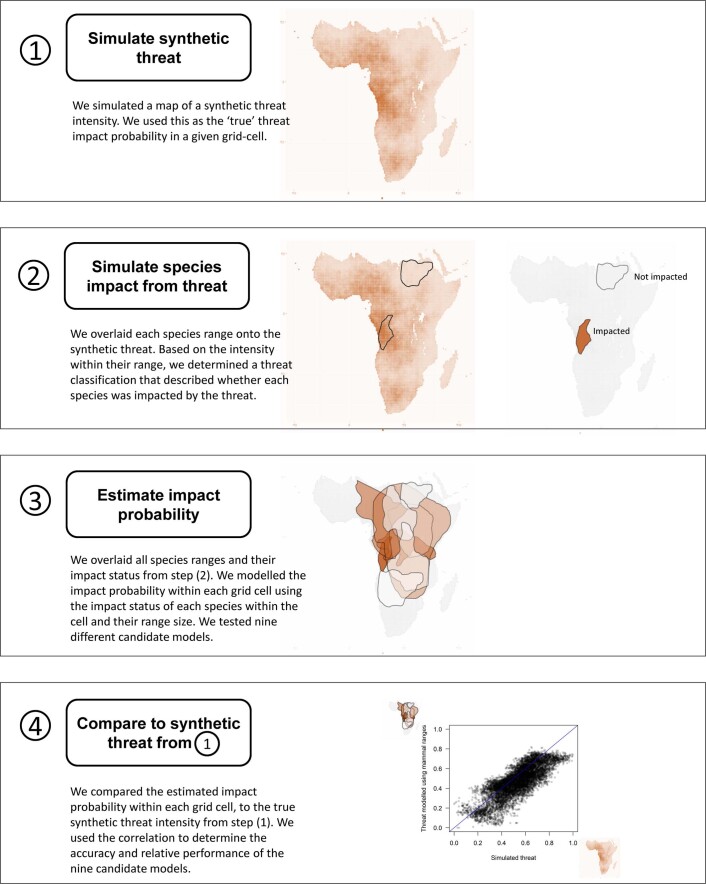
Framework for model generation. Step one involves the generation of an artificial simulated threat likelihood surface. In step 2 real range-maps are overlaid this surface and for each range, a category of either “affected” or “not affected” is assigned based on the values of the threat surface. Several different approaches based on median, mean, max was tested. In step three the ranges with the binary threat categorization are modelled using a series of probabilistic models. In step 4 the models from step 3 are correlated with the original simulated threat surface to identify the modelling approach that best re-produced the simulated surface. Once the best model is identified we progressed to validate it against two independent datasets.

**Extended Data Fig. 2:**
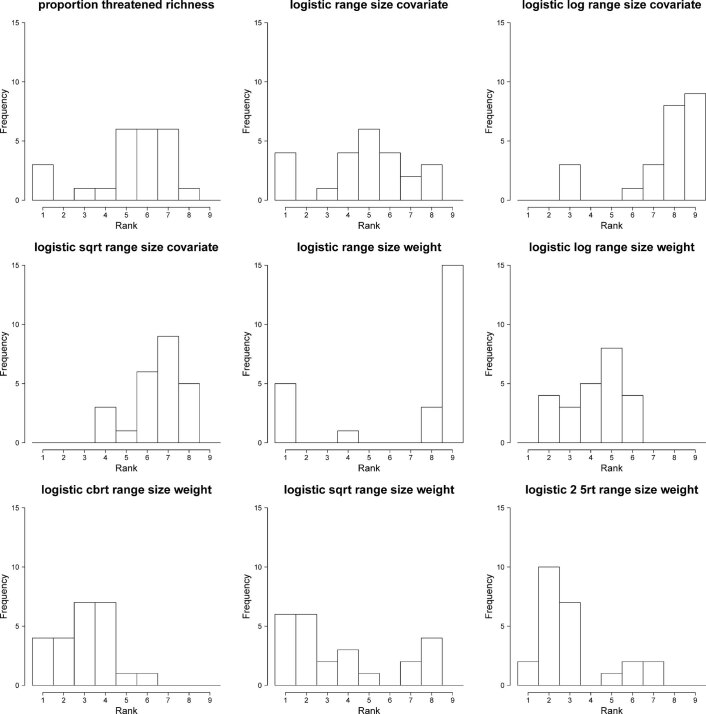
Rank frequency distributions for each of the models evaluated against simulated maps of threat intensity with different spatial autocorrelation (SAC) structures, (Low SAC r = 0.3, Medium SAC – r = 0.05, High SAC – r =  1 × 10^-4^, Very high SAC – r = 1 × 10^-6^) and uncertainties of knowledge used to generate threat codes (*C*_*0.25*_, *C*_*0.5*_, *C*_*0.75*_, *C*_*Uncertain-0.25*_, *C*_*Uncertain-0.5*_, *C*_*Uncertain-0.75*_).

Here we address these knowledge gaps by developing global maps for the six main threats affecting terrestrial amphibians, birds and mammals (23,271 species assessed by the IUCN Red List): (1) agriculture, (2) hunting and trapping, (3) logging, (4) pollution, (5) invasive species (including pathogens such as chytrid), and (6) climate change^4^. To generate these maps, we use data from the thousands of species assessed in the Red List in a probabilistic framework that explicitly incorporates the spatial uncertainty introduced by knowing only that a species is affected somewhere in the range. Our approach is inspired by methods from citizen science, which face similar issues resulting from large quantities of data with unknown and varying precision of the individual observations. We first used a set of simulated threat maps, with the same properties as the Red List, to develop our model framework and assess the ability of different model parameterizations to reproduce our simulated threat data (Extended Data Fig. 1). After choosing the model that showed the highest concordance with the simulated data (Extended Data Fig. 2), we mapped the impact probability of each of the six threats using the actual Red List data. We then evaluated the predictions of the best-fit model against two independent data sources: one on threats assessed by experts within about 6,000 Key Biodiversity Areas (KBAs)^20^ and a dataset based on remotely sensed forest change^10^. Both evaluations showed that our predictions were consistent with empirical data (Supplementary Figs. 5–11), giving us confidence in the validity of our approach to mapping the impacts of threats at a global scale.

## Results and discussion

### Major threats across taxa and space

**Fig. 1:**
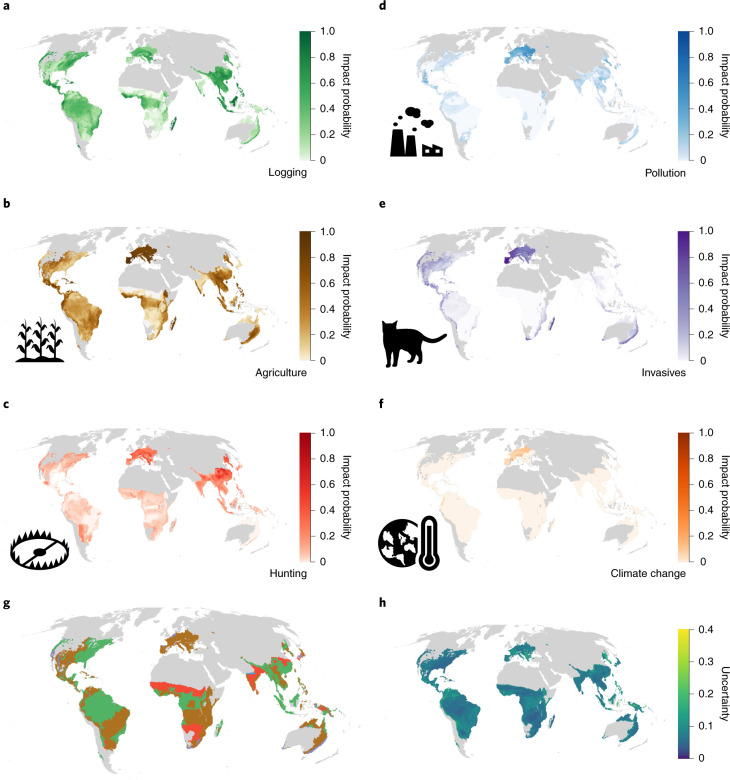
Probability of impact for amphibians. **a**–**f**, Probability that a randomly selected amphibian occurring in a 50 km × 50 km cell is impacted by logging (**a**), agriculture (**b**), hunting (**c**), pollution (**d**), invasive species (**e**) and climate change (**f**). Darker colours indicate higher probabilities. A value of 0 indicates that no species is affected, and 1.0 indicates that all species occurring are affected. Grey indicates areas with fewer than ten species for which the impact probability has not been estimated. **g**, The threat with the highest probability of impact in each cell. The colours correspond to the maximum colour scales in **a**–**f**. **h**, The variability of the estimates calculated by resampling the threat classes of each species on the basis of the proportion of Data Deficient species in a given cell (Methods).

**Fig. 2:**
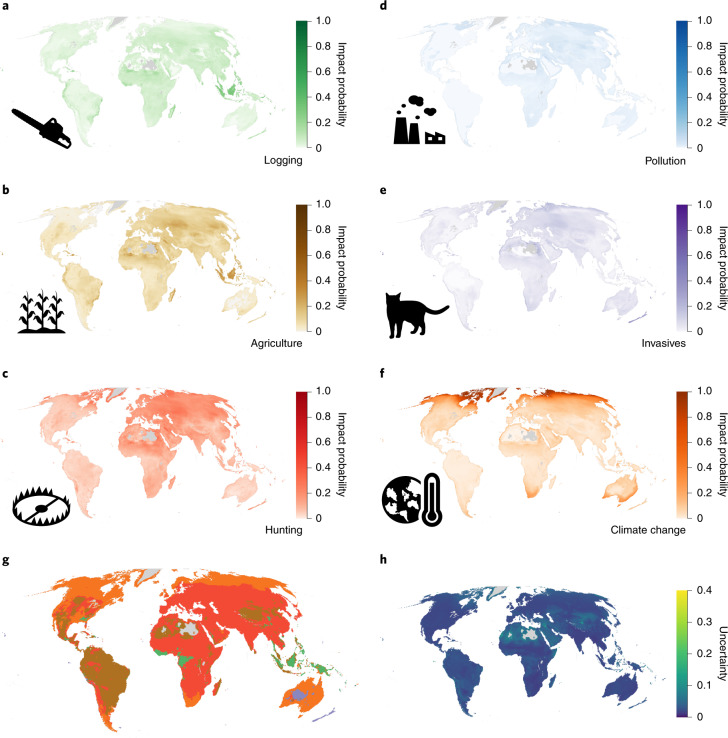
Probability of impact for birds. **a**–**f**, Probability that a randomly selected bird occurring in each 50 km × 50 km cell is impacted by logging (**a**), agriculture (**b**), hunting (**c**), pollution (**d**), invasive species (**e**) and climate change (**f**). Darker colours indicate higher probabilities. A value of 0 indicates that no species is affected, and 1.0 indicates that all species occurring are affected. Grey indicates areas with fewer than ten species for which the impact probability has not been estimated. **g**, The threat with the highest probability of impact in each cell. The colours correspond to the colour scales in **a**–**f**. **h**, The variability of the estimates calculated by resampling the threat classes of each species on the basis of the proportion of Data Deficient species in a given cell.

**Fig. 3:**
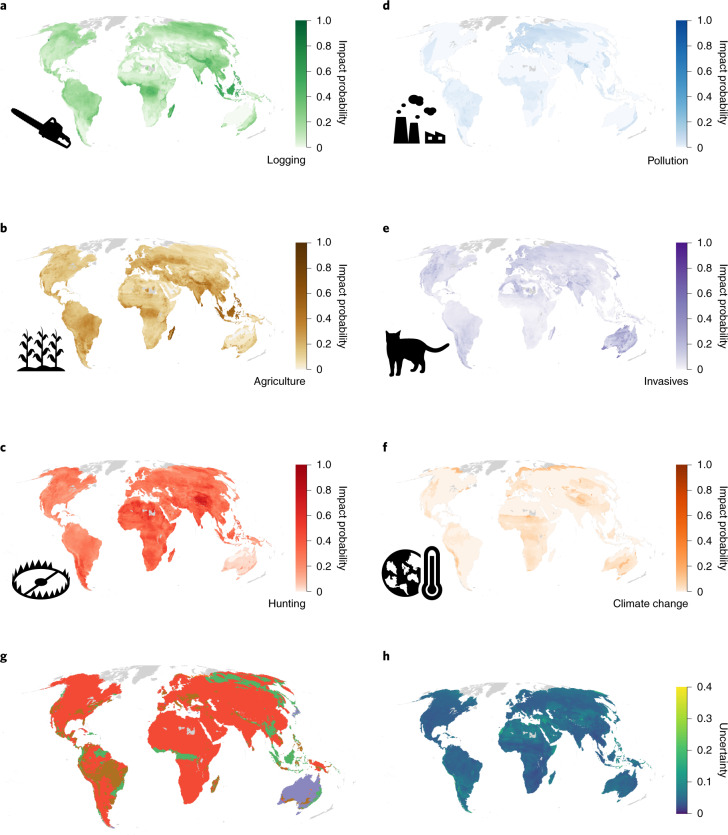
Probability of impact for mammals. **a**–**f**, Probability that a randomly selected mammal occurring in each 50 km × 50 km cell is impacted by logging (**a**), agriculture (**b**), hunting (**c**), pollution (**d**), invasive species (**e**) and climate change (**f**). Darker colours indicate higher probabilities. A value of 0 indicates that no species is affected, and 1.0 indicates that all species occurring are affected. Grey indicates areas with fewer than ten species for which the impact probability has not been estimated. **g**, The threat with the highest probability of impact in each cell. The colours correspond to the colour scales in **a**–**f**. **h**, The variability of the estimates calculated by resampling the threat classes of each species on the basis of the proportion of Data Deficient species in a given cell.

**Extended Data Fig. 3:**
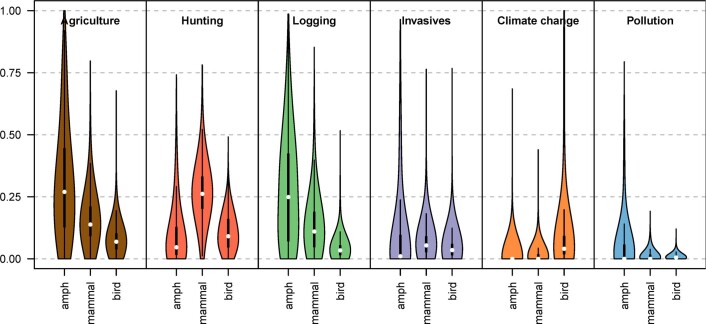
Distributions of the pixel occurrence likelihood values for the six threats and for each taxonomic group, arranged in descending order of mean impact probability across taxa per threat.

Our method estimates the ‘impact probability’, which is the probability that a randomly selected species occurring in a given grid cell is impacted in that cell by a particular threat, while accounting for the spatial uncertainty inherent in the Red List data. Across the six threats, amphibians had higher average impact probabilities (median threat probability across cells (*M*), 0.11; 95% confidence interval (*I*_95%_), 7 × 10^−3^ to 0.42), followed by mammals (*M* = 0.10; *I*_95%_, 0.04 to 0.21) and birds (M = 0.05; *I*_95%_, 0.01 to 0.19). This accords with the higher overall extinction risk of amphibians^21^. The largest uncertainties in the estimated impact probabilities were observed in the Congo Basin for amphibians and across the Sahara and Central Asia for birds and mammals (Figs. 1h, 2h and 3h). For amphibians, agriculture was the most prevalent of any threat, having the highest probability of impact in 44% of the mapped area (Fig. 1g), while hunting and trapping was the most prevalent threat for birds (in 50% of the mapped range; Fig. 2g) and overwhelmingly for mammals (73% of the mapped range; Fig. 3g). Our findings support existing non-spatial assessments^5^ and policy syntheses^4^. There are sizeable continental areas in which there was a greater than 50% chance that any given amphibian, mammal or bird species was threatened by logging, hunting and trapping, agriculture, invasive species or climate change (between 1.6 and 10.8 million km^2^; Extended Data Fig. 3).

Southeast Asia, particularly the islands of Sumatra and Borneo, as well as Madagascar, exhibited high probabilities of impact across all threats and all taxa (Figs. 1–3). For amphibians, Europe stood out as a region with high impact probabilities, driven by a combination of agriculture, invasive species (typically chytrid fungus) and pollution (Fig. 1), while polar regions, the east coast of Australia and South Africa showed the highest impact probabilities for climate change, driven in particular by impacts on birds (Fig. 2). For mammals and birds, high probabilities of species being impacted by hunting and trapping were found across much of the terrestrial surface (Figs. 2 and 3).

Across all taxa, agriculture had the highest average impact probability, followed by hunting and trapping and then by logging (Extended Data Fig. 3), while the probability of being impacted by pollution was low in most parts of the terrestrial world. The probability of a species being impacted by invasive species was on average low for amphibians (M = 0.01; *I*_95%_, 2.3 × 10^−10^ to 0.65), mammals (M = 0.05; *I*_95%_, 1.7 × 10^−9^ to 0.21) and birds (M = 0.04; *I*_95%_, 8.4 × 10^−11^ to 0.13). However, this probability of impact was elevated in some locations for birds and amphibians. For birds, the higher probabilities were seen on the islands included in our models. For amphibians, they were located on the east coast of Australia, in the dry forests of Madagascar, in Europe and in North America, the latter being consistent with observational data on recorded chytrid outbreaks^22^.

Our approach is also able to highlight where knowledge gaps about species distributions and threats most influence the certainty of our predictions by including the proportion of Data Deficient species in our analyses. While not a perfect proxy for knowledge certainty, the proportion of Data Deficient species is likely to correlate with overall certainty in knowledge in a given region. It is therefore reasonable to assume that if particular regions are less well studied, there will also be less certainty about the distribution, conservation status and threats to species in that region. We show that the largest uncertainties in the estimated impact probabilities were observed in the Congo Basin for amphibians and across the Sahara and Central Asia for birds and mammals (Figs. 1h, 2h and 3h). These regions have previously been identified as data-poor^23^, and increased sampling would probably improve both our model predictions and our understanding of threats to species in these areas.

### Priorities for threat mitigation

**Fig. 4:**
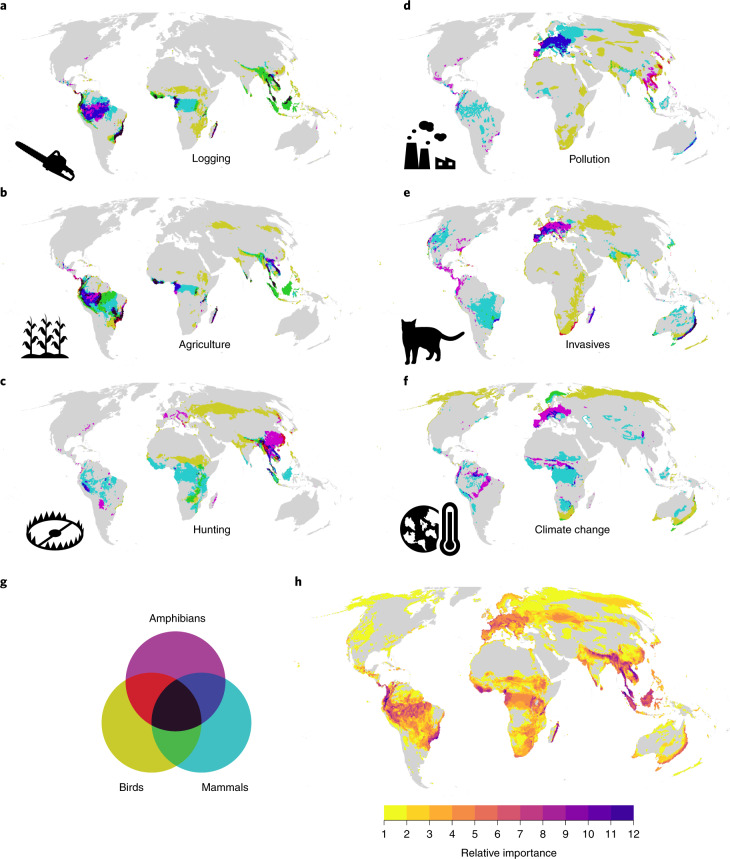
Global hotspots of threat. **a**–**f**, Global threat hotspots (90th percentile of risk, the product of the probability of impact and the species richness) for amphibians, birds and mammals for the six principal threats: logging (**a**), agriculture (**b**), hunting and trapping (**c**), pollution (**d**), invasive species and diseases (**e**), and climate change (**f**). The colours indicate whether an area falls within a threat hotspot for one or more taxon groups. **g**, Key for **a**–**f**. **h**, The relative importance of each pixel across species and threats. This is the number of times a pixel falls into a hotspot region for any taxon or threat, so pixels with higher values fall in the 90th percentile for many taxonomic groups and threats.

**Extended Data Fig. 4:**
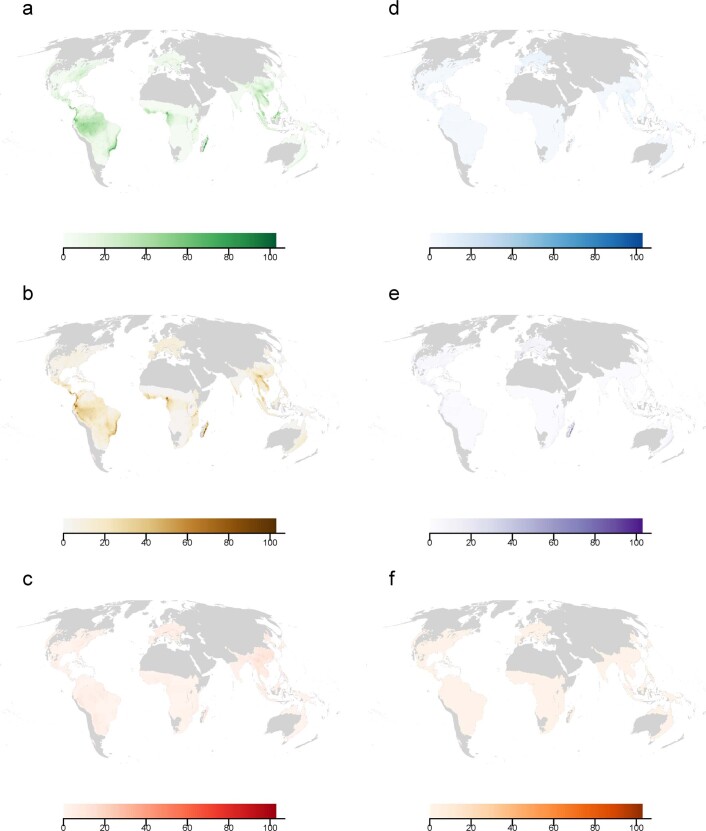
The expected number of amphibian species in each 50 × 50 km cell impacted by (**a**) logging, (**b**) agriculture, (**c**) hunting, (**d**) pollution, (**e**) invasive species, and (**f**) climate change. Darker colours indicate higher numbers. Grey indicates areas with fewer than 10 species for which the threat probability models were not calculated.

**Extended Data Fig. 5:**
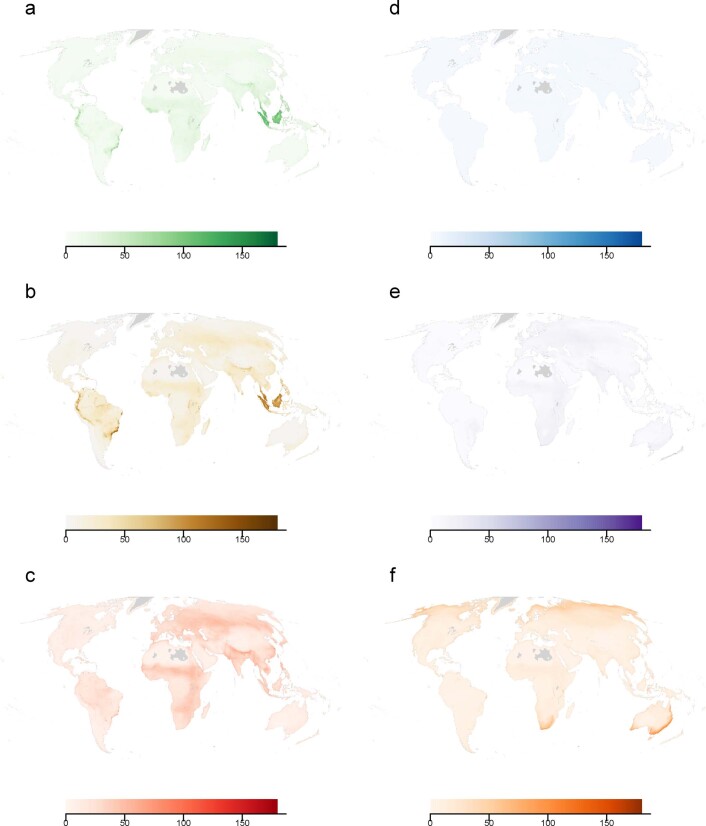
The expected number of bird species in each 50 × 50 km cell impacted by (**a**) logging, (**b**) agriculture, (**c**) hunting, (**d**) pollution, (**e**) invasive species, and (**f**) climate change. Darker colours indicate higher numbers. Grey indicates areas with fewer than 10 species for which the threat probability models were not calculated.

**Extended Data Fig. 6:**
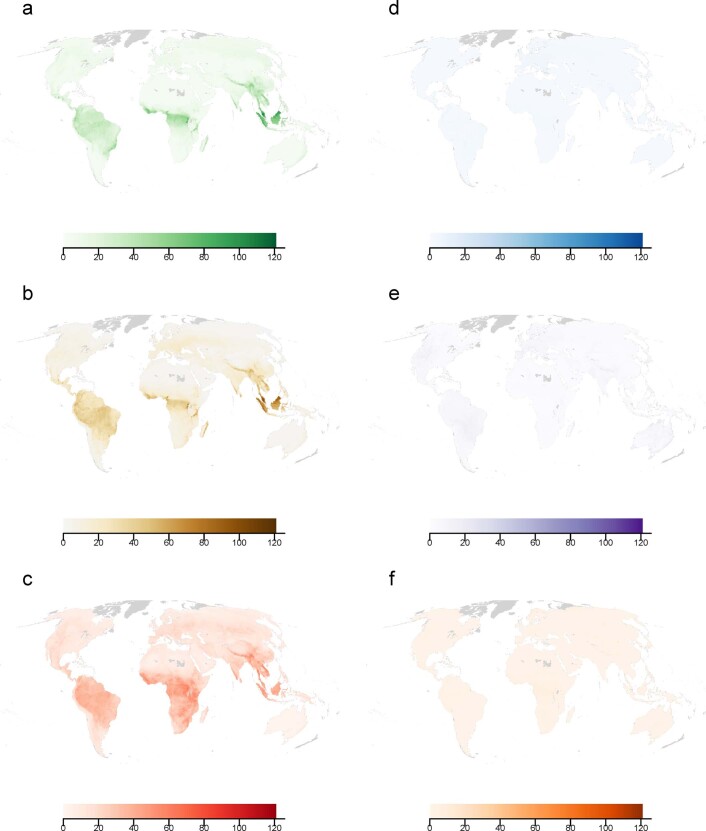
The expected number of mammal species in each 50 × 50 km cell impacted by (**a**) logging, (**b**) agriculture, (**c**) hunting, (**d**) pollution, (**e**) invasive species, and (**f**) climate change. Darker colours indicate higher numbers. Grey indicates areas with fewer than 10 species for which the threat probability models were not calculated.

To identify areas of priority for threat mitigation, it is necessary to combine the estimates of the probability that a threat impact occurs with information on the spatial pattern of biodiversity importance. We therefore developed conservation risk maps for each threat by multiplying our probability of impact with species richness (Extended Data Figs. 4–6). For each threat and taxonomic group, we then identified hotspot areas as the top decile (Fig. 4). Given the resolution of the Red List data and that of our maps (50 km × 50 km), our maps are not intended for guiding local conservation action but illustrate overall patterns of conservation priorities for mitigating threats to biodiversity across Earth.

Hotspots of the highest risk from agriculture, hunting and trapping, and logging were primarily located in the tropics. Conversely, hotspots of risk from pollution were found in Europe, driven by impacts on amphibians and mammals (Fig. 4a–f). Except for the Australian east coast, invasive species risk hotspots showed distinct patterns for the three taxa. Amphibians and mammals were particularly threatened in different parts of the New World and Europe, while hotspots of risk for birds were found on islands (consistent with existing syntheses^7,8,24^), along coastal areas and across eastern and southern Africa.

Hotspots of risk for different taxa rarely overlapped, and overall, 50% of the terrestrial surface was identified as a hotspot of risk from at least one threat for one taxonomic group (Fig. 4h). High-priority areas for threat mitigation were identified as the Himalayas, Southeast Asia, the east coast of Australia, the dry forest of Madagascar, the Albertine Rift and Eastern Arc Mountains in eastern Africa, the Guinean forests of West Africa, the Atlantic Forest, the Amazon basin and the Northern Andes into Panama and Costa Rica in South and Central America (Fig. 4h).

### Augmenting existing threat maps

Existing global threat maps estimate the extent of pressures or changes to the natural world such as land use, human settlements and infrastructure^11,12,25^. These maps capture the intensity of some of the most important human pressures on the environment, but they do not measure how these drivers and processes affect species and habitats^13^ and do not include all of the most important threats to biodiversity^5,26,27^. Our method, based on Red List data on threats to thousands of species, provides valuable complementary information. To assess the difference between maps based on drivers and processes and maps based on impacts on species, we compared our maps of impact probability with the latest version of the Human Footprint^11^.

**Fig. 5:**
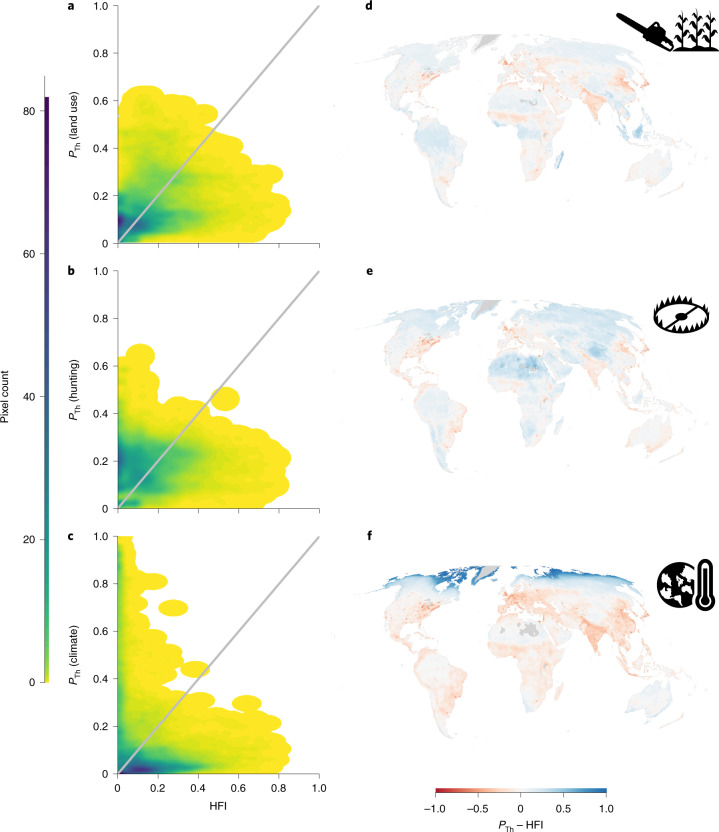
Comparison between probability of impact and pressure. **a**–**c**, Relationship between the Human Footprint Index (HFI) and the probability of threats (*P*_Th_) estimated from the Red List for amphibians, birds and mammals for land use (**a**) and for two threats that the HFI does not explicitly include: hunting and trapping (**b**) and climate change (**c**). The grey lines indicate a 1:1 linear relationship. **d**–**f**, Residuals from unity. Negative values (red) indicate where the standardized HFI is higher than *P*_Th_, and positive values (blue) indicate where *P*_Th_ is higher than the standardized HFI.

We first created a new composite land-use impact probability layer as the mean of agriculture and logging for each pixel, to better compare with the land-use component of the Human Footprint. We found a weak positive relationship between our measure of probability of impact from land use and the Human Footprint (Fig. 5a). However, there were discrepancies, with the Human Footprint generally showing lower pressures from land use in wilderness areas and higher pressures in urbanized areas compared with our impact probability map (Fig. 5d). This divergence was even more pronounced for hunting and trapping, with Human Footprint values relatively low across most of the tropical forests, where our maps suggest high impacts from hunting and trapping (Fig. 5b,e). The largest discrepancy was with climate change, for which some areas (especially in the Arctic) show a low Human Footprint but high impacts from climate change (Fig. 5c,f). Our results indicate that current global descriptions of pressure potentially underestimate the impact of human threats to biodiversity, particularly in the most pristine areas that are likely to be of high importance for nature conservation^28–30^. However, given the constraints associated with the species-based threat assessment used in the Red List, it is also plausible that our approach could overestimate the probability of impact for areas that, in reality, have low levels of threat and might serve as refugia for species. Our findings thus suggest that multiple approaches are needed, traversing drivers, processes and effects to better understand the multifaceted nature of human pressures on biodiversity. Additionally, while our threat maps represent the impacts on extant species due to threats from human drivers, they omit impacts from pressures that have already led to extirpations or extinctions. For example, in Europe, where a large part of the original fauna has already been lost^31^, maps of accumulated drivers (such as the Human Footprint) might better represent the true extent of human impacts than the response of the remaining species to current threatening factors^32^.

### Filling important knowledge gaps

Our approach helps address important data and knowledge gaps in more direct measures of threats based on field assessments by using a globally consistent, robust, and high-quality dataset^16,33,34^. For hunting and trapping, pollution, and invasive species, all of which are implicated in dramatic population declines of native species around the world^7,35–37^, our approach provides in some instances the only way of mapping their impacts on biodiversity at regional to global scales^9^. Even for threats for which remotely sensed maps of human activity exist (for example, agriculture and forest loss), our maps add additional information on where species seem to be adversely impacted by these activities. Regional analyses have also included information about species distributions to account for where threats are likely to affect most individuals^38^, but while valuable, such analyses still assume that threats are uniformly likely across the species range. Our results show that patterns of impact often differ from patterns of occurrence of threatening processes or the number of species affected by a given threat^39^. This discrepancy in part relates to current threat representations omitting some types of threats (for example, ‘empty forest syndrome’ resulting from pervasive hunting and trapping in apparently pristine forests^6,40,41^). Additionally, the effect of a threat varies with the specific context, so the same intensity of a threatening process can have different impacts in different places or on different species. For example, forest loss affects a larger proportion of species in Southeast Asia, where little primary forest is left, than in the Amazon, where substantial forest remains despite high rates of loss in both places^10^.

Our analysis and maps do not cover any plant or invertebrate groups, many of which are severely impacted by multiple threats^42–44^ and whose hotspots of diversity and conservation importance do not always overlap with those of terrestrial vertebrates^45^. Our work is also limited in terms of its representation of freshwater taxa. Additionally, our threat representation estimates the probability of a random species in a given location being affected by a threat. While we believe that this is closer to measuring the impact than mapping the drivers of threats, it does not capture the severity of the impact^46^. Thus, while our maps show that invasive alien species are not affecting very large numbers of species overall, the native species affected are often undergoing rapid population declines as a consequence^8,47,48^, particularly on many oceanic islands^7,8,24^. We acknowledge that it is possible that expert predispositions may influence assessments of some threats to some species on the Red List. However, the Red List assessment process is explicitly designed to mitigate this risk by ensuring that assessments are grounded in evidence from peer-reviewed and other vetted sources, properly documented, applied in a consistent fashion and subjected to independent review (see the Supplementary Information for a full description of the Red List assessment process).

### Improving future threat mapping

The current biodiversity crisis derives from current levels of action and resources being insufficient or misaligned to mitigate and reverse the increase in human pressures on the environment^3,49^. Thus, while the ultimate objective of conservation is to preserve biodiversity, understanding and addressing threats to nature is essential to ensure that action is targeted at the places most in need. Our maps provide an important step towards a more complete understanding of the distribution and impact of threats. However, this does not suggest that these maps cannot be improved. Indeed, a key strength of our approach is that it demonstrates a new way forward. The maps can help stimulate and inform models of how biodiversity is currently being impacted by a broader range of human activities than is typically considered. They can also help inform future red-listing assessments by providing a more systematic understanding of potential threats within the ranges of focal species. Additionally, other sources of data on anthropogenic pressures on biodiversity (such as from acoustic monitoring, camera traps, drones and satellite imagery) will be critical to help augment and improve our maps and develop more robust statistical synthesis of the impacts of threats to biodiversity. There is substantial potential for these maps to drive conservation science and policy. However, given the resolution of the maps and the precision of the underlying data, we caution against using these maps to guide local conservation action. Their value is in illuminating global patterns and demonstrating an approach to mapping threat impacts as well as informing decisions within the context of international policy processes such as the Convention on Biological Diversity and Sustainable Development Goals, recognizing that understanding where different threats impact terrestrial vertebrate species is essential for designing effective conservation responses^15^.

## Methods

### Species-level data

Species range maps were derived from BirdLife International and NatureServe^50^ and the IUCN^51^. The threat data were from the IUCN Threats Classification Scheme (Version 3.2), which contains 11 primary threat classes and almost 50 subclasses^52^. In Red List assessments, assessors assign those threats that impact the species. For birds, the scope of the impact is also recorded categorically as the percentage of the species population that the threat impacts (unknown, negligible, <50%, 50–90% or >90%) and the severity, describing the scale of the impact on population declines: unknown, no decline, negligible declines, fluctuations, slow but significant declines (<20% over ten years or three generations), rapid declines (20–30%) or very rapid declines (>30%).

### Model development approach

We designed our analytical framework with three considerations in mind. First, the threat location information is limited: for each species, the data only describe whether a species is threatened by a given activity anywhere within its range (data on the timing, scope and severity of threats are available only for birds and are not spatially explicit). Second, we wanted to compare the spatial patterns of threat against independent data on spatial distributions of human activities. Third, for many activities, the relationship between human activity (for example, hunting or invasive species and diseases) and biodiversity response is poorly understood. We therefore chose not to incorporate known patterns of human activity as explanatory variables in our models.

In the absence of global datasets on the spatial patterns of the impact probability of each threat, we used a simulation approach to develop our models and assess the ability of different model parameterizations to reproduce our simulated threat. This process had four steps (Extended Data Fig. 1).

#### Simulated threat intensity maps

First, we simulated a continuous synthetic threat across sub-Saharan Africa. The concept behind this is that a credible model should be able to reproduce a ‘true’, synthetic threat pattern on the basis of information comparable to that available in the Red List. To test this, we generated a set of synthetic, continuous surfaces of threat intensity with different levels of spatial autocorrelation and random variation (Supplementary Fig. 1). This was achieved by taking a grid of 50 km × 50 km (2,500 km^2^) pixels across the Afrotropic biogeographic realm (i.e., sub-Saharan Africa). Threat intensity was modelled as a vector of random variables, **Z**, one for each pixel *i*, generated with a correlation structure given by the distance matrix between points weighted by a scalar value, *r*, indicating the degree of correlation (equations (1–3)). Four values of *r* were used: 1 × 10^−6^, which yields very strong autocorrelation; 1 × 10^−4^, which yields strong autocorrelation; 0.05, which yields moderate autocorrelation; and 0.3, which produces a low-correlation, localized pattern (Supplementary Fig. 1). The model included the following equations:

1 $${\mathbf{Z}}(r) = U^{\mathrm{T}}{\mathrm{Norm}}\left( {n,0,1} \right)$$

2 $$W = UU^{\ast}$$

3 $$W = {\mathrm{e}}^{\left( { - rD} \right)}$$

where *r* is a scalar determining the degree of spatial autocorrelation (as *r* decreases, the autocorrelation increases), *D* is the Euclidean distance matrix between each pair of pixels, *W* is the matrix of weights for the threat intensity, *U* and U^*^ are the upper triangular factors of the Choleski decomposition of *W* and its conjugate transpose, *U*^T^ is the transpose of *U* and *n* is the number of pixels.

We chose the Afrotropic biogeographic realm (sub-Saharan Africa) as our geography within which to develop the modelling approach because it permitted more rapid iterations than a global-scale simulation while also retaining characteristics of importance for the model evaluation such as strong environmental gradients and heterogeneity in species richness. However, for the simulation, no information from the geography or overlapping species ranges was used, except the spatial configuration of the polygons. Thus, the use of the Afrotropic realm was purely to avoid generating thousands of complex geometries for the purpose of the simulation. Using a real geography and actual species ranges ensures that our simulation contains conditions that are observed in reality (for example, areas of high and low species richness also observed in the real world). We took the simulated threat maps generated through this process to be our ‘true’ likelihood of a randomly drawn species that occurs in that location being impacted by the synthetic threat (Supplementary Fig. 1).

#### Simulating the red-listing process

Second, we wanted to simulate the red-listing process whereby experts evaluate whether a threat is impacting a species on the basis of the overall threat intensity within its range. For this, we used the range maps for all mammal species in Africa and assigned a binary threat classification (that is, affected or not affected) to each species on the basis of the values of the synthetic threat within each species’ range. We assumed that the binary assessment of threat for a species is based on whether the level of impact across a proportion of its range is judged as significant. This step was intended to replicate the real red-listing process, where assessors define threats that impact the species on the basis of an assessment of the information available on threatening mechanisms and species responses. In practice, this was done by overlaying the real range maps for mammals over the four simulated threat surfaces and assessing the intensity of synthetic threat within each species range map. We wanted to assign species impacts considering that species will be more likely to be impacted if a greater part of their range has a high threat intensity. Understanding how to set a threshold for what intensity would constitute sufficient threat to be assessed as affected is a complicated exercise. We thus tested three thresholds to capture different assumptions. These thresholds were chosen after discussion with leading experts on the red-listing process. More specifically, we calculated the 25th, 50th and 75th percentiles of threat intensity across pixels within the species range. We then used a stochastic test to convert these quantiles to binary threat class, *C*. For each species, we produced a set of ten draws from a uniform distribution bounded by 0 and 1. If over half of the draws were lower than the threat intensity quantile, the species was classified as threatened for that percentile.

**Extended Data Fig. 7:**
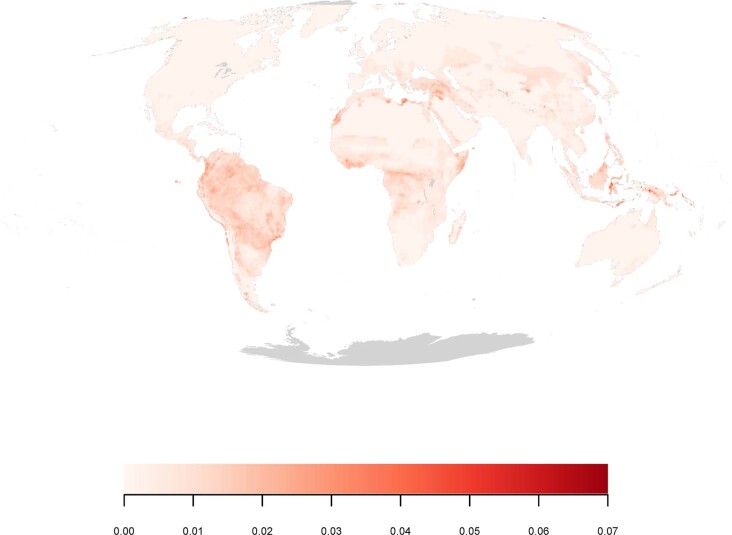
Map of the proportion of Data Deficient amphibian, bird or mammal species relative to the total number of species from these taxa occurring in each cell.

The above simulation assumes perfect knowledge of the threat intensities across the species range, which might not always be the case in the actual red-listing process. In real life, certain areas within species ranges are less well known for a suite of different reasons. To incorporate some uncertainty about the knowledge of the red-listing experts about the ‘true’ threat intensity, we constructed a layer to describe the spatial data uncertainty associated with the Red List. This aspect was intended to simulate the imperfect knowledge of the simulated ‘Red List assessors’. This layer was calculated as the proportion of species present in a given location that are categorized as Data Deficient—in other words, there is insufficient information known about the species to assess its extinction risk using the IUCN Red List Criteria (Extended Data Fig. 7). Then, when calculating the 25th, 50th and 75th percentiles of threat intensity across each range, we weighted this calculation by one minus the proportion of Data Deficient species, so that more uncertain places (those with a greater proportion of Data Deficient species) contributed less to the calculation than locations where knowledge was more certain. These were then converted to a binary threat class accounting for uncertainty in expert knowledge among the simulated ‘assessors’, *C*_Uncertain_, using the same stochastic process described above for the calculation of *C*.

This step produced, for each species, a threat classification analogous to the threat classification assigned by experts as part of the IUCN Red List process. Six sets of threat classifications were produced for each synthetic threat surface, on the basis of the 25th, 50th and 75th percentiles with perfect (*C*_0.25_, *C*_0.5_ and *C*_0.75_) or uncertain (*C*_Uncertain-0.25_, *C*_Uncertain-0.5_ and *C*_Uncertain-0.75_) spatial knowledge.

#### Model formulation and selection

Third, using all species polygons with assigned threat assessments from step 2 (that is, affected or not affected), we fitted nine candidate models and predicted the estimated probability of impact for each grid cell. Then, in a fourth step, we compared the predicted probabilities of impact produced in step 3 with the original synthetic threat maps created in step 1 to test the predictive ability of our models.

The Red List threat assessment does not contain information on where in the range the impact occurs. Therefore, a species with a very small range provides higher spatial precision about the location of the impact, whereas a species with a large range may be impacted anywhere within a wide region. To address this lack of precision in the impact location, we took the area of each species range to serve as a proxy for the spatial certainty of the impact information. The certainty that a species was impacted or not impacted in a given cell depended on its range size, *R*. The models we evaluated therefore incorporated *R* in different ways (Supplementary Table 1).

The models were fitted as a binomial regression with a logit link function. For each pixel, the model predicts the probability of impact, *P*_Th_—in other words, the probability that if you sampled a species at random from those that occur in that pixel, the species would be impacted by the activity being considered. To account for uncertainties in the simulation of the threat assessment process (thresholds for impact and perfect or imperfect knowledge), models were fitted to the six sets of threat codes (*C*_0.25_, *C*_0.5_, *C*_0.75_, *C*_Uncertain-0.25_, *C*_Uncertain-0.5_ and *C*_Uncertain-0.75_), and the root mean squared error (RMSE) was calculated between *P*_Th_ and the simulated threat intensity, **Z**(*r*), for each value of *r*. For each simulation, we ranked the different models according to their model fit as measured by the RMSE. We assessed these ranks across all simulations and sets of threat codes. We evaluated the models on the basis of the ranks of RMSE, across the threat code sets and threat intensity maps. Rank distributions for each model are shown in Extended Data Fig. 2, and the results from these models are shown in Supplementary Tables 1 and 2.

All models were correlated (Pearson’s *r*^2^ > 0.5), albeit with some variation between model types and across the simulation parameters (Supplementary Fig. 2). However, some models had greater predictive accuracy when evaluated using the RMSE. The top four ranking models were, in order of decreasing summed rank, (1) inverse of cube root of range size as a weight, (2) inverse 2.5 root of range size as a weight, (3) inverse square root of range size as a weight and (4) inverse natural logarithm of range size as a weight. The fact that these four models showed good model fit suggests that the best model structure had a measure of range size as a weight but that the model was not particularly sensitive to the transformation of range size.

The best-fitting model across the range of simulation parameters was an intercept-only logistic regression where the response variable was the binary threat code (1 = threatened, 0 = not threatened) for each species in the pixel and where the inverse cube root of the range size of each species was used as a weight. The model was concordant across the set of simulated datasets with a relationship that was predominantly linear with *r*^2^ between 0.47 and 0.7, depending on simulation parameters for **Z**(*r*) in 0.05, 10^−4^ and 10^−6^, centred around unity and with the RMSE ranging between 0.129 and 0.337 depending on simulation parameters (Supplementary Figs. 2 and 3). The choice of the inverse cube root range size weight was based on the performance of this against eight other model types (Supplementary Fig. 4 and Supplementary Table 1).

We conducted a decomposition of variance in model performance using a binomial regression model, with RMSE as the dependent variable and model type, knowledge level and autocorrelation structure as the independent factorial variables. This showed that knowledge about the threats underlying each species range and how that threat information is used in the assessment explained the vast majority (94.7%) of the variation in RMSE outcomes (Supplementary Fig. 4).

For birds, further information on the scope of the threat was available as an ordinal variable describing the fraction of range that the threat covers. We explored the use of scope in our models but concluded that to avoid arbitrary decisions about the scope of non-threatened species (where they are either not threatened anywhere or threatened in only a small part of their range), and for consistency with other taxonomic groups, we would model birds using the same model structure as used for mammals and amphibians (see the Supplementary Methods for further details).

### Mapping probability of impact

Once the best-performing model was identified using the simulated data, we then used this model on the actual Red List threat and range data to develop threat maps. This model produced threat maps for each taxonomic group (amphibians, birds and mammals) of the probability of impact, *P*_Th_, for each individual threat. For a given pixel, threat and taxonomic group, this estimates the probability that a randomly sampled species with a range overlapping with that pixel is being impacted by the threat, while taking into account spatial imprecision in the Red List data.

Threat maps were generated using range map data and threat assessments from the IUCN Red List^18^. We intersected range maps for 22,898 extant terrestrial amphibians (*n* = 6,458), birds (*n* = 10,928; excluding the spatial areas within the range that are associated with ‘Passage’—where the species is known or thought very likely to occur regularly during relatively short periods of the year on migration) and mammals (*n* = 5,512; including those with uncertain ranges) with a global 50 km × 50 km (2,500 km^2^) resolution, equal-area grid for the terrestrial world. This provided, for each 50 km × 50 km pixel, a list of the species whose range overlapped it, along with the associated range size of each species. For each pixel and taxonomic group (amphibians, birds and mammals) independently, we then modelled the probability of impact, *P*_Th,Activity_ (for example, *P*_Th,Logging_ for logging, *P*_Th,Agriculture_ for agriculture or *P*_Th,Pollution_ for pollution), for each of the six threats: agriculture, hunting and trapping, logging, pollution, invasive species and diseases, and climate change. We focused on these as the six main threats as defined by the Intergovernmental Science-Policy Platform on Biodiversity and Ecosystem Services^4^, but our methodological framework is flexible and could be expanded to other threats in the IUCN classification^19^. We used only taxonomic groups with a sufficiently high total number of species and where they have been comprehensively assessed so that potential biases associated with the groups of species prioritized by experts are avoided.

### Calculating uncertainties for the threat probability

**Extended Data Fig. 8:**
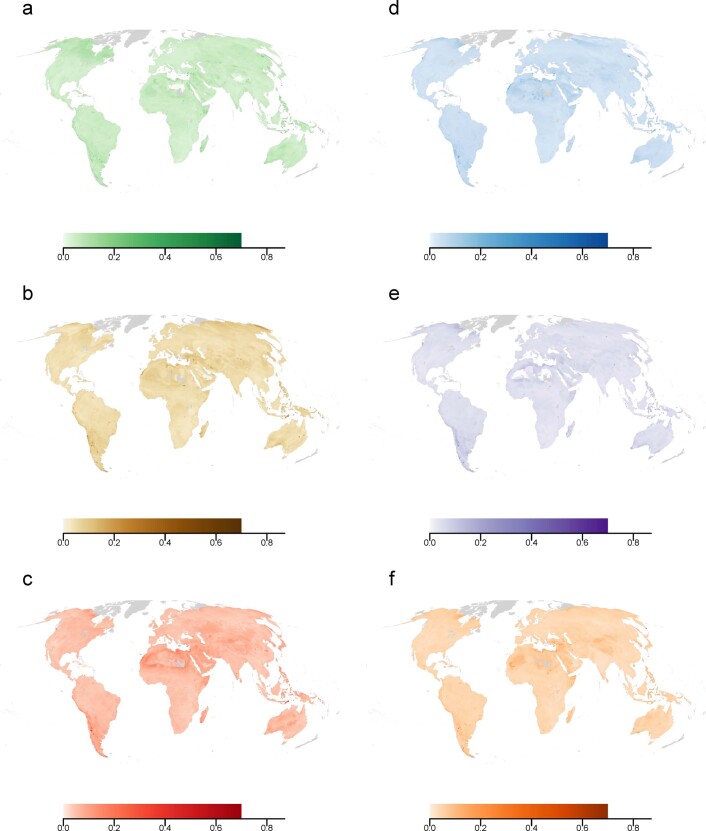
Uncertainty associated with estimated threat probabilities for mammals in response to (**a**) logging, (**b**) agriculture, (**c**) hunting, (**d**) invasive species, (**e**) pollution and (**f**) climate change. Uncertainty is calculated as the 95-per cent range of estimated threat probabilities from 100 draws of threat codes taking into account uncertainty of knowledge about each species informed by the proportion of Data Deficient species across each species range.

**Extended Data Fig. 9:**
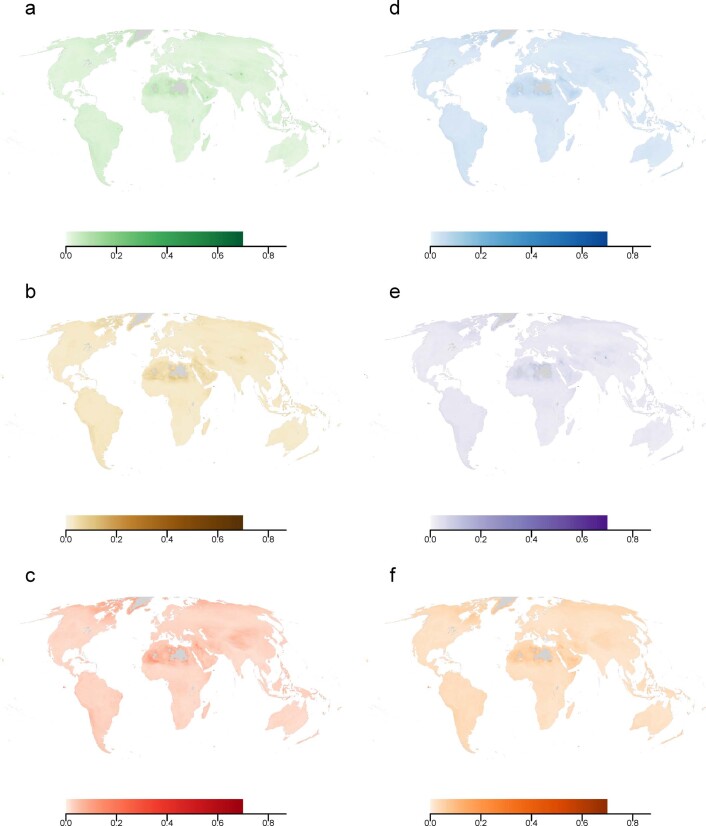
Uncertainty associated with estimated threat probabilities for birds in response to (**a**) logging, (**b**) agriculture, (**c**) hunting, (**d**) invasive species, (**e**) pollution and (**f**) climate change. Uncertainty is calculated as the 95-per cent range of estimated threat probabilities from 100 draws of threat codes taking into account uncertainty of knowledge about each species informed by the proportion of Data Deficient species across each species range.

**Extended Data Fig. 10:**
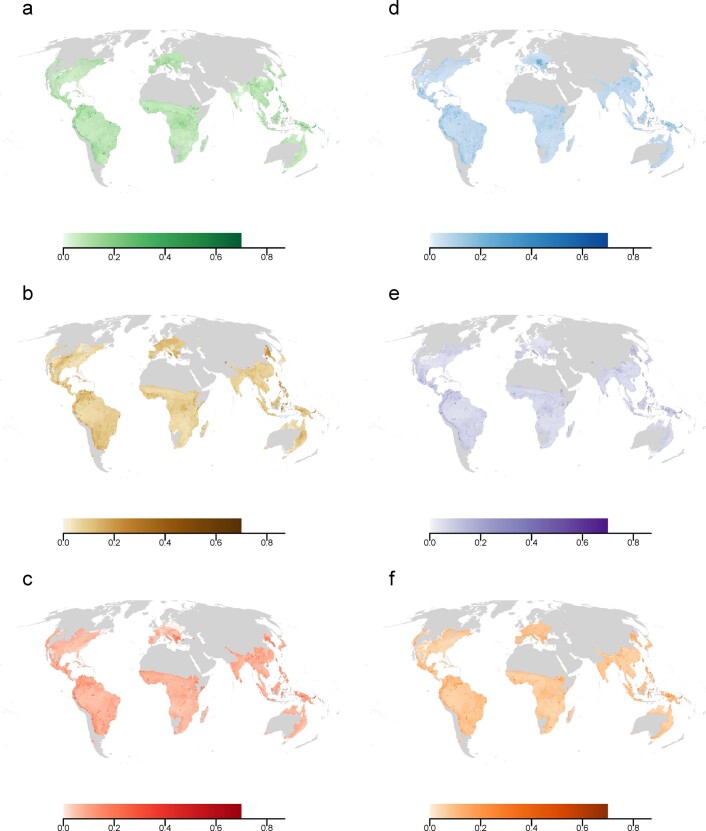
Uncertainty associated with estimated threat probabilities for amphibians in response to (**a**) logging, (**b**) agriculture, (**c**) hunting, (**d**) invasive species, (**e**) pollution and (**f**) climate change. Uncertainty is calculated as the 95-per cent range of estimated threat probabilities from 100 draws of threat codes taking into account uncertainty of knowledge about each species informed by the proportion of Data Deficient species across each species range.

We estimated a measure of uncertainty associated with our impact probability predictions using maps of the proportions of Data Deficient species in each cell within each taxonomic class (amphibians, birds or mammals) as a measure of knowledge certainty in that cell. The rationale for this approach is that places with more Data Deficient species with unknown threatened status should have greater uncertainty in the probability of impact. We therefore created greater variation in the data where there were more Data Deficient species. We used the knowledge-certainty map to probabilistically draw a sample of 100 threat codes for each species, on the basis of the median Data Deficiency across the species range. The random sample changed the species threat code with a probability related to the proportion of Data Deficient species within its range. If the median proportion of Data Deficient species was zero, then we assumed that there was a small probability (0.005) that the species could have been incorrectly coded. Where the median proportion was greater than zero, the probability increased linearly. So, for a species with 5% Data Deficient species within its range, the sample changed the species threat code with a probability close to 5%; if the median proportion was equal to 0.5, then the probability of the species being incorrectly assigned was equal to 0.5. We then fitted the impact probability model with each of the 100 species threat codes and generated a distribution of predicted threat probabilities in each grid cell, from which we took the 95% confidence intervals as the uncertainty estimates (Extended Data Figs. 8–10).

### Evaluating modelled threat patterns

We evaluated the spatial patterns of threat on the basis of the real Red List threat assessment data against empirical data in two independent ways. First, we compared the probability of impact from logging and agriculture combined within forested biomes (that is, corresponding to remotely detected forest loss, which we refer to as the probability of impact from forest loss, *P*_Th,Forest-loss_) with data on forest cover change^10^. Forest cover change was aggregated from their native 30 m × 30 m (900 m^2^) resolution pixels to our 50 km × 50 km resolution pixels using Google Earth Engine. For each 50 km × 50 km pixel, we calculated the total area lost between 2000 and 2013 and the area lost as a proportion of the area in 2000. We restricted our analysis to forested biomes: (1) tropical and subtropical moist broadleaf forests, (2) tropical and subtropical dry broadleaf forests, (3) tropical and subtropical coniferous forests, (4) temperate broadleaf and mixed forests, (5) temperate coniferous forests and (6) boreal forests/taiga, following the World Wildlife Fund’s ecoregions classification^53^. The relationship between forest loss and the probability of impact from forest loss as captured by agriculture and logging overall showed a significant positive correlation: *P*_Th,Forest-loss_ increased with increasing forest cover loss (*P* < 1 × 10^−5^, Supplementary Fig. 5) but also showed some nuances.

Second, we evaluated threat levels against threat for about 6,000 KBAs assessed by specialists through a rigorously tested and standardized approach developed by Bird Life International^20^. For a given activity, we calculated the median of predicted impact probabilities within each KBA and then grouped these KBA estimates by KBA severity class. On average, *P*_Th_ was higher in KBAs assessed as having a high severity of threat from an activity than in KBAs classed as having low threat. Significant relationships (*P* < 0.05, Wilcoxon test) were found in one or more taxonomic groups for logging, agriculture, hunting and climate change. For both evaluations, we conclude that the modelled spatial patterns of threat were consistent with expectations from the empirical data (Supplementary Figs. 5–11). We subsequently shared threat maps with taxon-specific experts from the Red List assessment groups, who qualitatively reviewed the patterns and assessed them as consistent with expert knowledge. Further details on the evaluation methods can be found in the Supplementary Methods.

We suggest that the broad concordance with empirical data in two independent evaluations demonstrates that the maps of impact probability are sufficiently plausible to underpin the findings of this study. We provide a framework that can easily be updated with future versions of the IUCN data, and we also stress that our approach should be viewed as an initial attempt to map patterns of threat impacts, which should be used iteratively to guide the collection of new data and improvement of the mapping approaches used.

### Comparing threat occurrence likelihoods and the HFI

HFI data for the year 2009 were taken from Venter et al.^11^. The native resolution of the index was 1 km^2^, so we calculated the mean HFI in each 50 km × 50 km pixel used in our analysis. The HFI was standardized to lie within the bounds 0 and 1 by dividing by the maximum HFI value (50).

We compared the standardized, averaged HFI values to the predicted likelihood of threat occurring from land use change, hunting and trapping, and climate change. For land use change, we combined agriculture and logging by calculating the unweighted mean threat occurrence likelihood across taxa for these two threats. For hunting and trapping, we took the mean threat occurrence likelihood across taxa, while for climate change we used the predicted threat occurrence likelihood for birds.

To plot the spatial relationship between HFI and mean threat occurrence likelihood, we used a two-dimensional kernel density estimator (MASS package^54^) to estimate the variation in the density of pixels for a given HFI and mean threat occurrence likelihood.

### Reporting Summary

Further information on research design is available in the Nature Research Reporting Summary linked to this article.

## Supplementary information

Supplementary Information Supplementary Methods, Figs. 1–14 and Tables 1–5.

Reporting Summary

Peer Review Information

## Data Availability

The data on range maps are freely available at https://www.iucnredlist.org/resources/spatial-data-download and http://datazone.birdlife.org/species/requestdis. The IUCN threat classification assessment data can be downloaded using the Red List API (http://apiv3.iucnredlist.org/api/v3/docs) or on request from the IUCN’s Global Species Programme Red List Unit (redlist@iucn.org). Other data are freely available using citations in the manuscript.

## Code availability

The code is available from the GitHub repository (https://github.com/mikeharfoot/ThreatMapping).

## References

[1] Steffen W, Grinevald J, Crutzen P, McNeill J (2011). The Anthropocene: conceptual and historical perspectives. Phil. Trans. R. Soc. A.

[2] Barnosky AD (2011). Has the Earth’s sixth mass extinction already arrived?. Nature.

[3] Tittensor DP (2014). A mid-term analysis of progress toward international biodiversity targets. Science.

[4] *Intergovernmental Science-Policy Platform on Biodiversity and Ecosystem Services, 2019* (The IPBES Global Assessment on Biodiversity and Ecosystem Services, Intergovernmental Science-Policy Platform on Biodiversity and Ecosystem Services, 2019).

[5] Joppa LN (2016). Filling in biodiversity threat gaps. Science.

[6] Benítez-López A, Santini L, Schipper AM, Busana M, Huijbregts MAJ (2019). Intact but empty forests? Patterns of hunting-induced mammal defaunation in the tropics. PLoS Biol..

[7] Early R (2016). Global threats from invasive alien species in the twenty-first century and national response capacities. Nat. Commun..

[8] Spatz DR (2017). Globally threatened vertebrates on islands with invasive species. Sci. Adv..

[9] Wilson K (2005). Measuring and incorporating vulnerability into conservation planning. Environ. Manage..

[10] Hansen MC (2013). High-resolution global maps of 21st-century forest cover change. Science.

[11] Venter O (2016). Sixteen years of change in the global terrestrial human footprint and implications for biodiversity conservation. Nat. Commun..

[12] Ellis EC, Ramankutty N (2008). Putting people in the map: anthropogenic biomes of the world. Front. Ecol. Environ..

[13] Balmford A (2009). Capturing the many dimensions of threat: comment on Salafsky et al. Conserv. Biol..

[14] Raiter KG, Possingham HP, Prober SM, Hobbs RJ (2014). Under the radar: mitigating enigmatic ecological impacts. Trends Ecol. Evol..

[15] Tulloch VJD (2015). Why do we map threats? Linking threat mapping with actions to make better conservation decisions. Front. Ecol. Environ..

[16] Brooks TM (2015). Harnessing biodiversity and conservation knowledge products to track the Aichi targets and Sustainable Development Goals. Biodiversity.

[17] Juffe-Bignoli D (2016). Assessing the cost of global biodiversity and conservation knowledge. PLoS ONE.

[18] The IUCN Red List of Threatened Species Version 2019-3 (IUCN, 2019); https://www.iucnredlist.org

[19] Salafsky N (2008). A standard lexicon for biodiversity conservation: unified classifications of threats and actions. Conserv. Biol..

[20] *Monitoring Important Bird Areas: A Global Framework* Version 1.2. (BirdLife International, 2006).

[21] Hoffmann M (2010). The impact of conservation on the status of the world’s vertebrates. Science.

[22] Hof C, Araujo MB, Jetz W, Rahbek C (2011). Additive threats from pathogens, climate and land-use change for global amphibian diversity. Nature.

[23] Mammides C (2016). Increasing geographic diversity in the international conservation literature: a stalled process?. Biol. Conserv..

[24] Holmes ND (2019). Globally important islands where eradicating invasive mammals will benefit highly threatened vertebrates. PLoS ONE.

[25] Sanderson EW (2002). The human footprint and the last of the wild. Bioscience.

[26] Secretariat of the Convention on Biological Diversity *Global Biodiversity Outlook 5* (Convention on Biological Diversity, 2006).

[27] Hulme PE (2018). Protected land: threat of invasive species. Science.

[28] Watson JEM (2018). Protect the last of the wild. Nature.

[29] Mittermeier RA (2003). Wilderness and biodiversity conservation. Proc. Natl Acad. Sci. USA.

[30] Di Marco M, Ferrier S, Harwood TD, Hoskins AJ, Watson JEM (2019). Wilderness areas halve the extinction risk of terrestrial biodiversity. Nature.

[31] Barnosky AD, Koch PL, Feranec RS, Wing SL, Shabel AB (2004). Assessing the causes of Late Pleistocene extinctions on the continents. Science.

[32] Yackulic CB, Sanderson EW, Uriarte M (2011). Anthropogenic and environmental drivers of modern range loss in large mammals. Proc. Natl Acad. Sci. USA.

[33] Butchart SHM (2005). Using Red List indices to measure progress towards the 2010 target and beyond. Phil. Trans. R. Soc. B.

[34] Rodrigues ASL (2014). Spatially explicit trends in the global conservation status of vertebrates. PLoS ONE.

[35] Di Minin E (2019). Identifying global centers of unsustainable commercial harvesting of species. Sci. Adv..

[36] Dirzo R (2014). Defaunation in the Anthropocene. Science.

[37] Ripple WJ (2016). Bushmeat hunting and extinction risk to the world’s mammals. R. Soc. Open Sci..

[38] Evans MC (2011). The spatial distribution of threats to species in Australia. BioScience.

[39] Schipper J (2008). The status of the world’s land and marine mammals: diversity, threat, and knowledge. Science.

[40] Redford KH (1992). The empty forest. Bioscience.

[41] Stokstad E (2014). The empty forest. Science.

[42] McCullough DG, Work TT, Cavey JF, Liebhold AM, Marshall D (2006). Interceptions of nonindigenous plant pests at US ports of entry and border crossings over a 17-year period. Biol. Invasions.

[43] Theoharides KA, Dukes JS (2007). Plant invasion across space and time: factors affecting nonindigenous species success during four stages of invasion. N. Phytol..

[44] Pyšek P (2008). Geographical and taxonomic biases in invasion ecology. Trends Ecol. Evol..

[45] Jung, M. et al. Areas of global importance for terrestrial biodiversity, carbon, and water. *Nat. Ecol. Evol.*10.1038/s41559-021-01528-7 (2020). 10.1038/s41559-021-01528-7 34429536

[46] Hulme PE (2014). Greater focus needed on alien plant impacts in protected areas. Conserv. Lett..

[47] Lydeard C (2004). The global decline of nonmarine mollusks. Bioscience.

[48] McGeoch MA (2010). Global indicators of biological invasion: species numbers, biodiversity impact and policy responses. Divers. Distrib..

[49] Coad L (2019). Widespread shortfalls in protected area resourcing significantly undermine efforts to conserve biodiversity. Front. Ecol. Environ..

[50] *Bird Species Distribution Maps of the World* (BirdLife International, NatureServe, 2017).

[51] *Red List of Threatened Species* Version 2017.3 (IUCN, 2017).

[52] *IUCN–CMP Threats Classification Scheme* Version 3.2.20 (International Union for the Conservation of Nature, Conservation Measures Partnership, 2019).

[53] Olson DM (2001). Terrestrial ecoregions of the world: a new map of life on Earth. Bioscience.

[54] Venables, W. N. & Ripley, B. D. *Modern Applied Statistics with S* 4th edn (Springer, 2002).

