# Supplementary material for: Using the IUCN Red List to map threats to terrestrial vertebrates at global scale
Source: Nat Ecol Evol. 2021 Aug 30;5(11):1510–9. doi: 10.1038/s41559-021-01542-9 (PMC8560638; doi:10.1038/s41559-021-01542-9)
Supplement: Supplementary file 1 — Supplementary Methods, Figs. 1–14 and Tables 1–5. [file 41559_2021_1542_MOESM1_ESM.pdf]

nature portfolio

https://doi.org/10.1038/s41559-021-01542-9

Supplementary information

Using the IUCN Red List to map threats to terrestrial vertebrates at global scale

In the format provided by the authors and unedited

Supplementary Methods: Towards a global map of threats to species

Authors: Mike Harfoot, Alison Johnston Andrew Balmford, Neil D. Burgess, Stuart H. M. Butchart, Maria P. Dias, Carolina Hazin, Craig Hilton-Taylor, Mike Hoffmann, Nick J. B. Isaac, Lars Iversen, Charlotte L. Outhwaite, Piero Visconti, and Jonas Geldmann

Correspondence to: Jonas Geldmann (jgeldmann@sund.ku.dk) and Mike Harfoot (Mike.Harfoot@unep-wcmc.org)

There were three distinct activities involved in developing the modelling approach. First, we used simulated data to develop the modelling framework and to select the best performing model. Second, we evaluated our predictions of the spatial pattern of the probability of impact from forest loss (combining agriculture and logging) against patterns of forest loss from the Global Forest Watch dataset[6]. Third we evaluated our predictions of probability of impact against independent estimate of threat occurrence from the World Database of Key Biodiversity Areas (KBAs) maintained by Bird Life International[7]. The methods for these activities are described in the main manuscript. This supplementary methods text provides details for certain aspects of the method not described in detail in the main text.

Assessing the use of threat scope: For birds, further information on the scope of the threat was available as an ordinal variable describing the fraction of range that the threat covers. Scope takes the following levels: >90%, 50 to 90%, <50%, negligible and unknown. For birds alone, we therefore simulated a threat coding process including the scope of the threat. We based this assessment simulation on the same simulated threat intensity layers, *Z(r)*, described above. We overlaid the simulated threat layer with bird range maps. The scope and threat code for a species was then calculated as follows. For each pixel within a species range compared the threat intensity value for the pixel against a set of 10 draws from a uniform distribution bounded by 0 and 1. Where the threat intensity was greater than the uniform draw then the species was considered to be impacted in that cell for that draw. We then calculated the median value across draws of the proportion of the species range that was covered by pixels classified as impacted and classified the species impact scope against the four categories described above.

The scope information regarding threat provides exactly the weight that should be applied to each species when considering how its binary impact code for a given threat contributes to the likelihood of impact in any given pixel within its range. We therefore fitted a logistic regression model with the structure $P_{Th} \sim 1$ weighted by the scope. When we evaluated the predicted likelihood of impact from this model type against the simulated threat maps, we did not find any increase in performance. Additionally, we found there is a challenge in how to handle species that are not threatened by the simulated activity. These are either not threatened anywhere within their range or are threatened in only a small part, but this has minimal impact on the threat status of the species. Where the species is not threatened anywhere, it should in theory receive a scope of 100%, however, doing this, leads to non-threatened species being weighted more highly than any threatened species, whose scopes are always less than 100% when modelling the probability of impact. So, to avoid arbitrary decisions about the scope of non-threatened species (where they are either not threatened anywhere or threatened in only a

small part of their range), and for consistency with other taxonomic groups, we modelled birds using the same model as used for mammals and amphibians – logistic regression using the inverse cube root range size as a weight. However, we see great potential in the threat-classification process used for birds that better capture the spatial extent and intensity

Evaluating modelled threat patterns

We performed two further evaluations against independent datasets to understand the performance of the modelling approach against real world threat activities or threat assessment. First we compared P_{Th} for forest loss against the observational data on forest loss. We chose this human activity because it is one of the best understood. The spatial patterns of forest loss were informed by forest cover change[6].

Second, for all activities, we compared the estimated impact probabilities to categorical assessments of threat severity for over 6,000 Key Biodiversity Areas (KBAs) for which local experts had assessed the intensity of threats using a consistent method and the same threat-classification scheme as ours.

Comparison threat likelihood with forest cover change: We first combined the probabilities of impact from agriculture and logging to better represent the threatening processes captured by remote sensed forest cover loss. Forest cover change was aggregated from their native 30x30 m (900 m^2) resolution pixels to our 50×50 km resolution pixels using Google Earth Engine. For each 50×50 km pixel we calculated the total area lost between 2000 and 2013 and the area lost as a proportion of the area in 2000. We restricted our analysis to forested biomes: a) Tropical and subtropical moist broadleaf forests, b) Tropical and subtropical dry broadleaf forests, c) Tropical and subtropical coniferous forests, d) Temperate broadleaf and mixed forests, e) Temperate Coniferous Forest and f) Boreal forests / Taiga, following WWFs ecoregions classification[12].

The relationship between forest loss and the probability of impact from forest loss as captured by agriculture and logging overall showed a significant positive correlation (Supplementary Figure 5) but also showed some nuances. First, most pixels experience little proportional forest loss, whilst many of these pixels with small proportional losses showed high probabilities of impact for mammals and amphibians (Supplementary Figure 5). To test the relationship between the probability of impact and forest loss, we fitted a binomial regression model of $P_{Th,\ Logging}$ against proportional forest loss as the predictor. The results of this indicated a significant ($p < 0.01$) positive relationship between $P_{Th,\ Logging}$ and proportional forest loss for all taxa.

While the relationship is not perfect, we note that it does provide evidence for consistency between the two datasets. Further, the imperfect relationship is not surprising for reasons in addition to our model being highly parsimonious. Loss of forest is not analogous to being threatened by forest clearing, and the relationship between forest loss and impact is highly complex[13-16]. Thus, a perfect linear relationship was never the expectation. We therefore mapped the residuals for the correlation to investigate potential patterns in where the relationship was strong and weak. This map of the residuals (Supplementary Figures 6-8) showed patterns that were consistent with our expectations. Overall, the fact that the residuals of model correspond to real-world patterns, provides additional validation to our method. The residuals of our models were larger and negative in areas corresponding to current deforestation frontiers (e.g. the eastern edge of the Amazon and the edge of forested Sumatra). This shows

that in areas of intense on ongoing forest clearing, remote-sensed maps provide a higher intensity estimate of the current clearing process, while our model on the other hand provides a clearer picture of threatening process from loss of forest being more pervasive than just where tree-cover is lost as of now – extending beyond the current clearing frontier.

Comparison with KBA data: which was obtained from BirdLife International and provided information on over 6,000 KBA sites, in total. Each KBA has a delineated boundary (polygon) and an assessment of threats affecting the key species for which the site was identified as a KBA. This assessment identifies the severity of impact using a four-step ordinal scale ("no or imperceptible deterioration", "slow but significant deterioration", "moderate to rapid deterioration" and "very rapid to severe deterioration"). The threat severity category describes how impactful the threat is in terms of how rapidly it drives declines in the species populations within the KBA. As such, it is the element of the assessment scoring that is most equivalent to what our method maps. For each of the human activity classes, KBAs can be grouped into their severity category. We then analysed the threat occurrence likelihoods predicted by the model across pixels intersecting with each KBA, severity class- human activity group. This was done for all six of the human activity classes.

We found that, with some variation, the probability of impact tended to increase with increasing impact severity category and this was more pronounced for the major threats of logging, agriculture and hunting (Supplementary Figure 9). Kendall rank correlation tests confirmed this – 16 out of 18 coefficients were weakly positive and all but three of those were significant (Supplementary Figure 10, $p < 0.05$).

As a second evaluation of model performance against the KBA data, we compared the different in predicted threat for KBAs with a high threat severity with those assessed as having a low severity. Since the KBA threat severity assessment categories use a four-step ordinal scale where it is difficult to assess the "distance" between intermediate levels, we chose to compare the threat probabilities of KBAs classed as having 'no' or 'slow' (no = "no or imperceptible deterioration" and slow = "slow but significant deterioration") threat impact severity with those classed as having 'moderate' or 'very rapid' severity (very rapid = "very rapid to severe deterioration" and moderate = "moderate to rapid deterioration").

As hypothesized, KBAs assessed to have a higher threat severity were projected by our models to have a higher threat likelihood than KBAs classed as having lower threat severity (Supplementary Figure 11). The positive difference between more impacted and less impacted KBAs was significant for all taxonomic groups and human activity classes with the exception of birds impacted by pollution and climate change, where the results were non-significant, and mammals impacted by invasive species and climate change, where the results were negative. The predicted threat to mammals from invasives had a negative correlation with the rank of KBA severity class (Supplementary Figure 10) whilst there was negligible difference and no significant correlation in the predicted threat from climate change across KBA severity classes.

There are several reasons why we do not expect our results to perform perfectly. In addition to the limitations in the Red List data and in the modelling approach documented in the simulation studies above, the KBA-threat severity assessment is not exactly equivalent to our threat likelihood. The classification of threat in the KBAs relates to threats within the site impacting the species for which the site qualifies as a KBA under the KBA criteria[17]. With these caveats in mind, we conclude that the comparison with KBA data also broadly supports the validity of our modelling approach.

The effect of range- and body-size: To assess whether there is a correlation between species sensitivity to the threat and range size, we looked at the relationship between the range-size for all species and the assigned threat code for each of the primary threat categories, to examine whether range-size was associated with threat. We subsequently did the same for body-size, a trait that is often linked to range size[18,19] and potentially renders species more susceptible to some threat [20].

Body mass data for mammals and birds were extracted from the EltonTraits v1 dataset[21]. Body mass data for 5,748 amphibians were extracted from AmphiBIO[22] using empirical masses (=2,011) or estimated (=3,737) using body length and allometric relationships[23]. We used data from Oliveira et al.,[22] and Santini et al.,[23] on snout to vent lengths and body mass to derive allometric relationships between SVL and body mass for anurans, caudatans, caecilians (order Gymnophiona). We fitted separate models for each order following the evidence in Santini et al., 2017 supporting fitting the orders separately. We did not include information on the habitat preferences for species since this was not available for most species for which SVL and body mass were available. Both SVL and body mass were log10 transformed prior to linear model fitting. Model statistics show that the models capture the data reasonably well ($r^2 > 0.6$; $p < 0.01$), despite the fact that there was limited data available for caecilians (Supplementary Table 3).

Overall, we found that there was an extremely small, but often significant ($p < 0.05$), effect of range size on the likelihood of being threatened by an activity. In the majority of cases this was negative showing that smaller ranging organisms were more likely to be threatened than large ranging counterparts, but this varied considerably across threat activities and taxa with no consistent clear pattern (Supplementary Figure 12, Supplementary Table 4). There was also a small, often significant, effect of body mass on threat likelihood, however, the direction was often in disagreement with the range size effect (Supplementary Figure 13, Supplementary Table 5). For example, species threatened by agriculture and logging were more likely to have a marginally smaller range size than those species assessed as not threatened. However, threatened species were more likely to have larger body size than those not threatened. This analysis suggests that the relationship between species traits and how species are threatened by human activities is both complex and variable, in agreement with previous literature[24,25], and therefore that it is challenging to parsimoniously incorporate such traits into a general model of threat likelihood.

Evaluation discussion

We have found the modelling results consistent with evaluation data, but to varying degrees across three independent methods of evaluation: first against simulated data, second against well the observed threatening activity of logging, and lastly against expert assessment of threat severity within protected areas. The best performing model had errors of 13-18% depending on the spatial autocorrelation structure of the threat process with greater error when the spatial autocorrelation is low. Therefore, the models are likely to perform better when the human activity forming the threating process (e.g. logging, agriculture or pollution) has higher spatial autocorrelation as opposed to activities that have spatially sporadic and limited extents.

The logistic regression model using the cube root of inverse range size as a weight, was better at explaining simulated threat patterns and performed better in evaluation against independent KBA threat assessment data, compared to a model using the proportion of species threatened by the activity. In addition to performing better, the model including weight addresses a

methodological weakness in the Red List threat assessment data, namely that the spatial information about where a given threat occurs within a species range.

That the results of what is an extremely parsimonious model show general consistency with what might be expected from empirical data, suggests that the models and their predictions are sufficiently plausible to underpin the analyses and conclusions of this study. Nonetheless there are a broad range of improvements that we envision for the future. The most important is to develop a hierarchical model structure that takes into account the spatial connection of threats, functional traits of species relevant to the threat process or alternatively phylogenetic relationships that might confer information about the correspondence of threat.

Supplementary Figure 1.

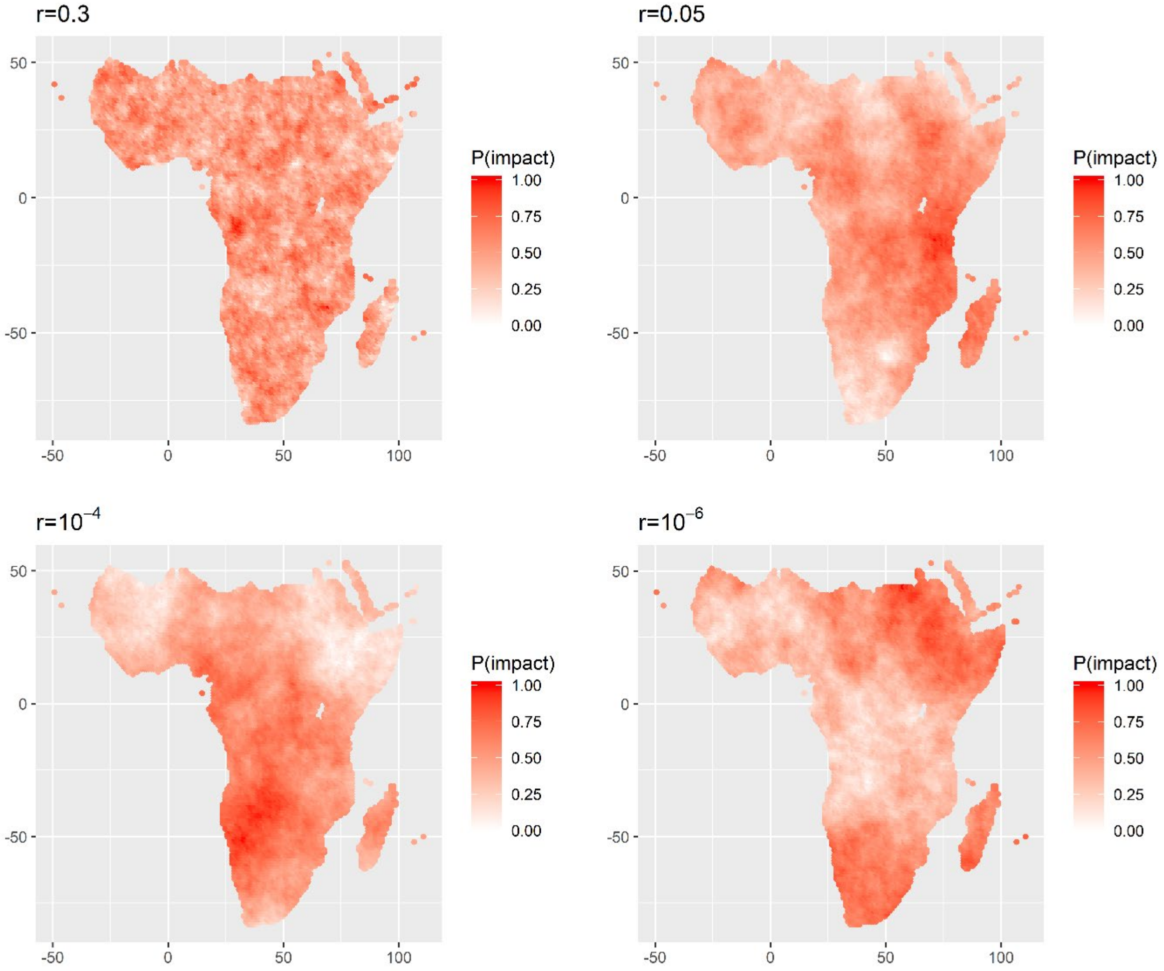

Supplementary Figure 1. Map of simulated threat intensity, *Z(r)*, with low (r = 0.3), medium (r = 0.05), high (r = 1×10^{-4}) and very high spatial autocorrelation structure (r = 1×10^{-6}).

Supplementary Figure 2.

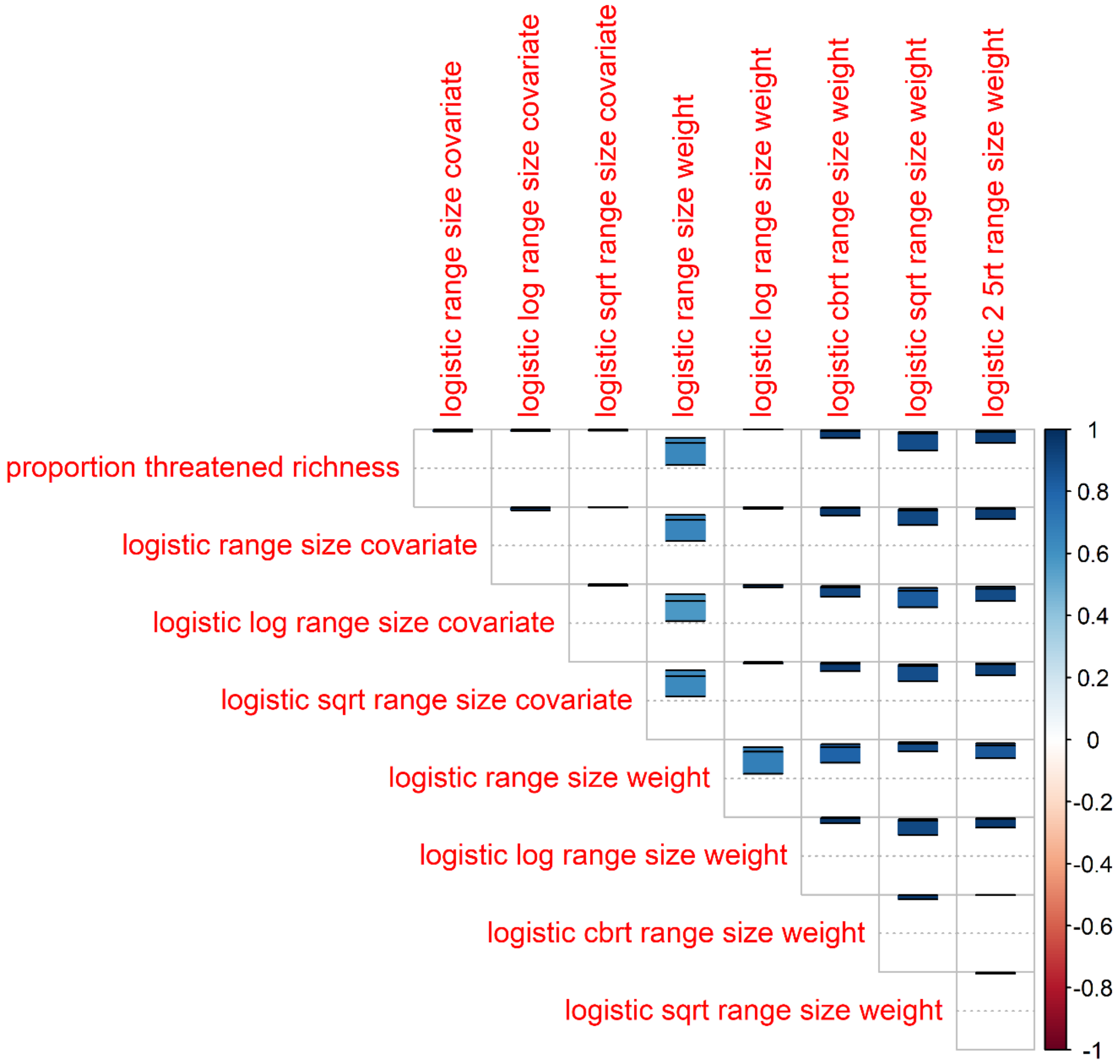

Supplementary Figure 2. Pearson correlation coefficient distribution for each of the models evaluated against simulated maps of threat intensity with different spatial autocorrelation (SAC) structures, (Low SAC r = 0.3, Medium SAC – r = 0.05, High SAC – r = 1×10^{-4}, Very high SAC – r = 1×10^{-6}) and uncertainties of knowledge used to generate threat codes ($C_{0.25}$, $C_{0.5}$, $C_{0.75}$, $C_{Uncertain-0.25}$, $C_{Uncertain-0.5}$, $C_{Uncertain-0.75}$).

Supplementary Figure 3.

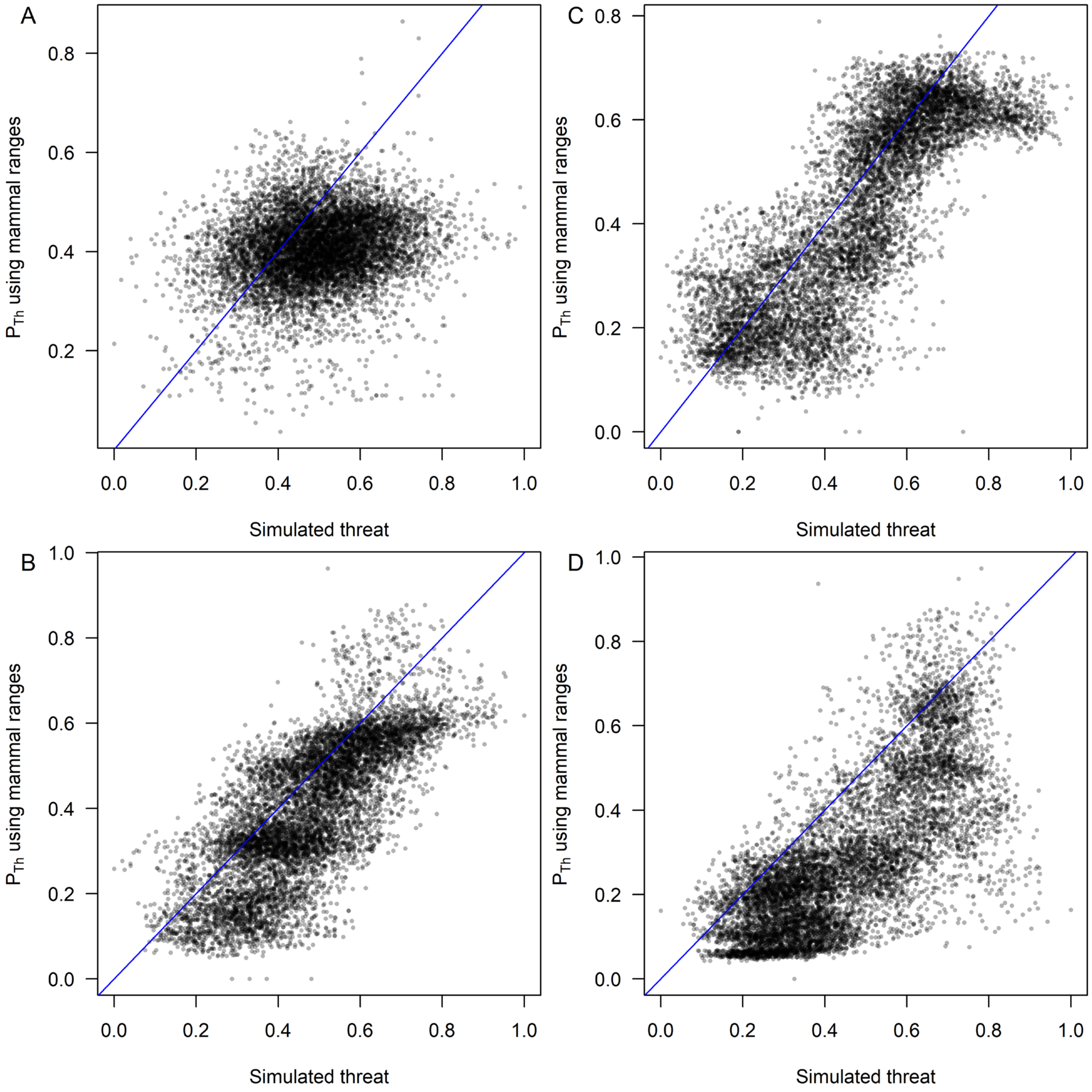

Supplementary Figure 3. Visualisations of the characteristic relationship between simulated threat and predicted probability of impact using the logistic regression model with inverse cube root range as a weight for $C_{Uncertain\text{-}0.5}$ and the four different map types, *$Z(r = 0.3)$ (A), $Z(r = 0.05)$ (B), $Z(r = 1\times10^{-4})$ (C) and $Z(r = 1\times10^{-6})$ (D)*. Points indicate pixels within the Afrotropic biogeographic realm and blue line indicates the 1:1 line.

Fig. S4.

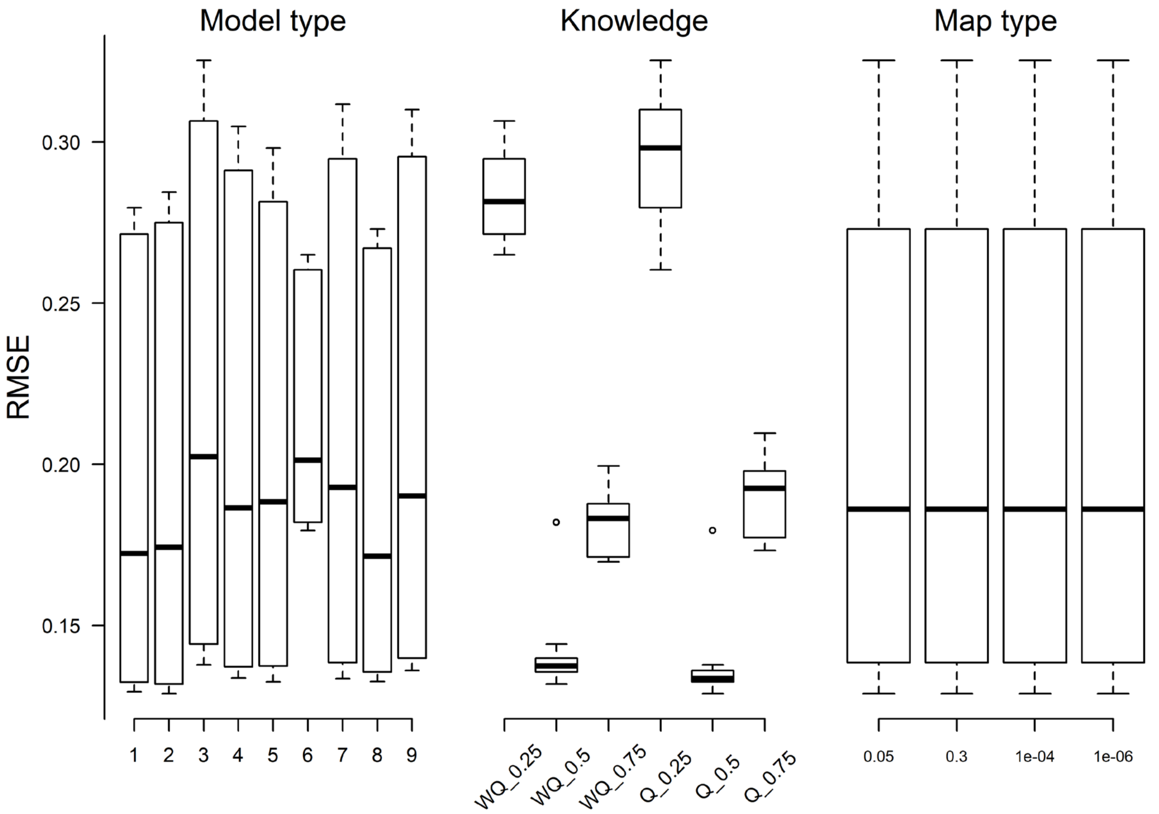

Supplementary Figure 4. Variance decomposition of square root mean squared error (RMSE) between model estimated threat probability and simulated maps of threat intensity with different spatial autocorrelation (SAC) structures, (P0.3 = Low SAC - r = 0.3; P0.05 = Medium SAC – r = 0.05; P1e-04 = High SAC – r = 1×10^{-4}; P1e-06 = Very high SAC – r = 1×10^{-6}) and uncertainties of knowledge used to generate threat codes (Q_0.25 = $C_{0.25}$, Q_0.5 = $C_{0.5}$, Q_0.75 = $C_{0.75}$, WQ_0.25 = $C_{Uncertain-0.25}$, WQ_0.5 = $C_{Uncertain-0.5}$, WQ_0.75 = $C_{Uncertain-0.75}$). Model numbers correspond to the models described in Supplementary Table 1.

Supplementary Figure 5.

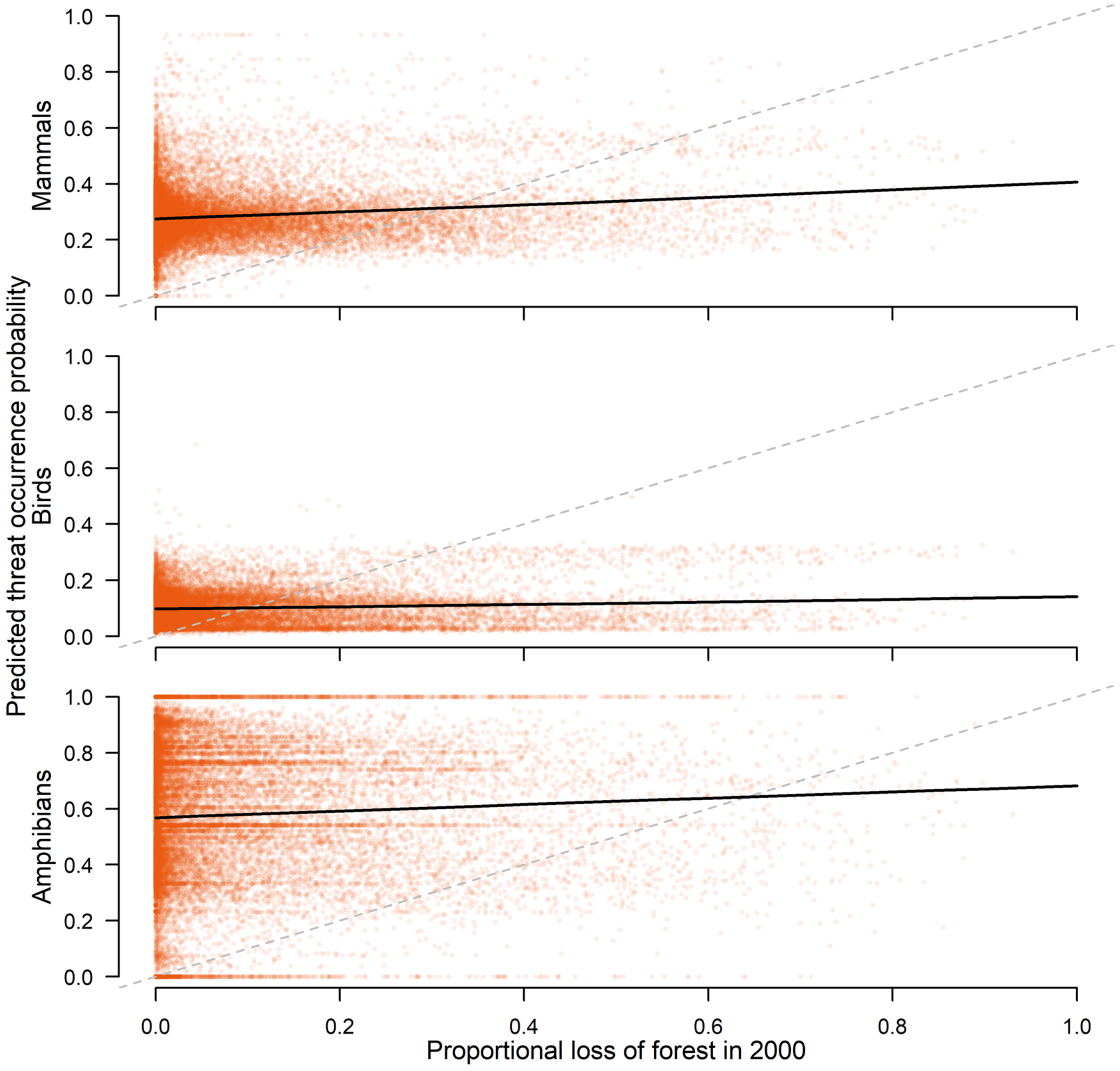

Supplementary Figure 5. The relationship between logging threat occurrence likelihood for mammals, birds and amphibians and the proportion of year 2000 forest cover lost by 2013, per 50×50 km pixel aggregated from Hansen et al. [6]. Points indicate pixels, solid black lines indicate the trend in threat probability with proportional forest loss from a binomial regression model, and the grey dashed line represents the 1:1 line. Slope coefficients were significant for all taxa ($p < 0.01$).

Supplementary Figure 6.

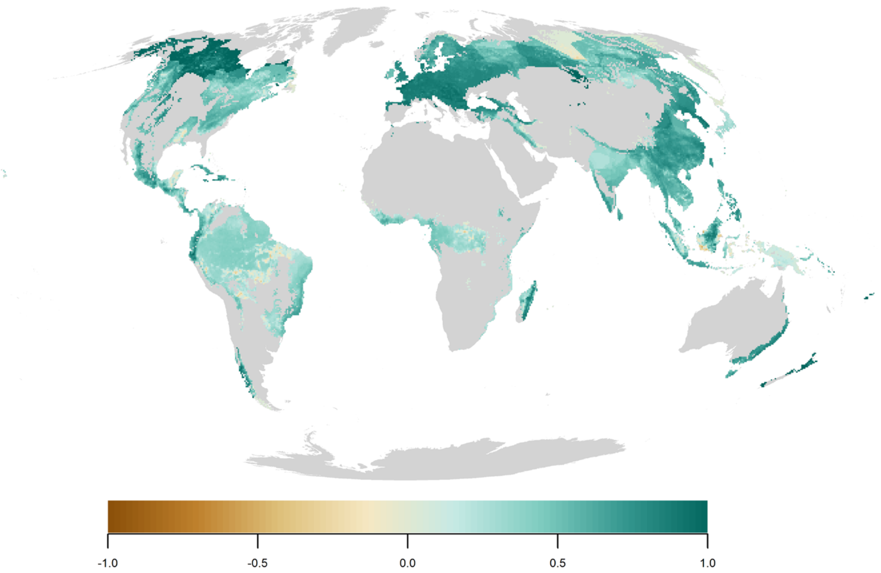

Supplementary Figure 6. Residuals of correlation between predicted likelihood of impact from agriculture and logging combined against remote-sensed forest loss for amphibians. Green values indicate locations where residuals are positive (i.e. our model of likelihood of impact predicts higher threat from deforestation than suggested by the rate of loss of remote-sensed forest cover). Patterns are consistent with expectations. Areas around the western and southern edge of the amazon as well as a southern frontier on Borneo show negative values consistent with very intense current forest loss. Comparatively, boreal North America and much of Europe, where impact are more related to agriculture but the rate of forest loss in absolute terms are lower, show positive residual.

Supplementary Figure 7.

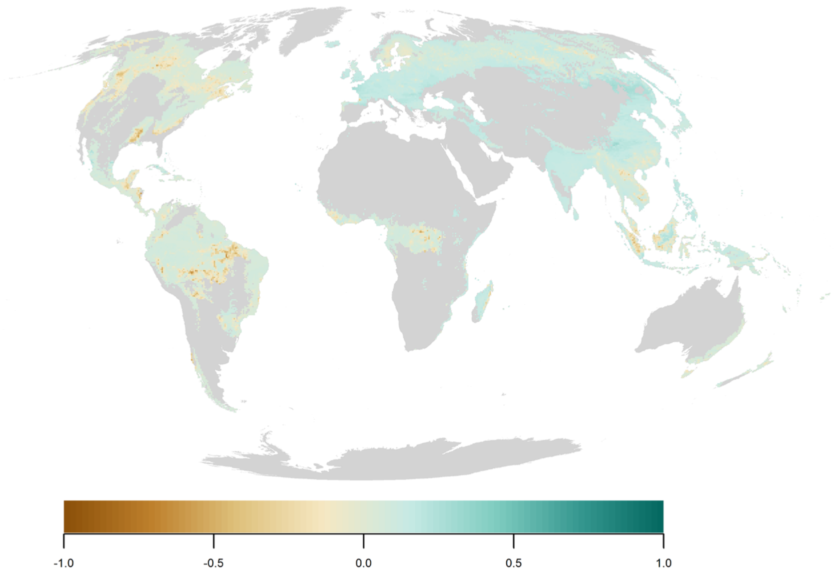

Supplementary Figure 7. Residuals of correlation between predicted likelihood of impact from agriculture and logging combined against remote-sensed forest loss for birds. Green values indicate locations where residuals are positive (i.e. our model of likelihood of impact predicts higher threat from deforestation than suggested by the rate of loss of remote-sensed forest cover). Patterns are consistent with expectations. Areas around the western and southern edge of the amazon, and the Congo basin frontier as well as a southern frontier on Borneo show negative values consistent with very intense current forest loss. Comparatively, much of Europe and India, where impact of forest loss are great, but the rate of forest loss in absolute terms are lower, show positive residual.

Supplementary Figure 8.

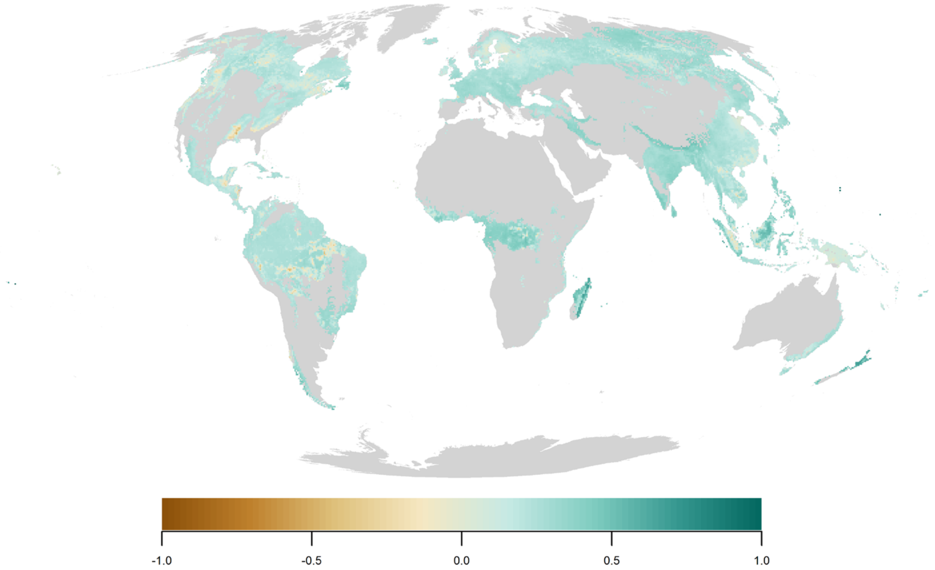

Supplementary Figure 8. Residuals of correlation between predicted likelihood of impact from agriculture and logging combined against remote-sensed forest loss for mammals. Green values indicate locations where residuals are positive (i.e. our model of likelihood of impact predicts higher threat from deforestation than suggested by the rate of loss of remote-sensed forest cover). Patterns are consistent with expectations. Areas around the western and southern edge of the amazon, and the Congo basin frontier as well as a southern frontier on Borneo show negative values consistent with very intense current forest loss. Comparatively, much of Europe and Madagascar, where impact of forest loss are great, but the rate of forest loss in absolute terms are lower, show positive residual.

Supplementary Figure 9.

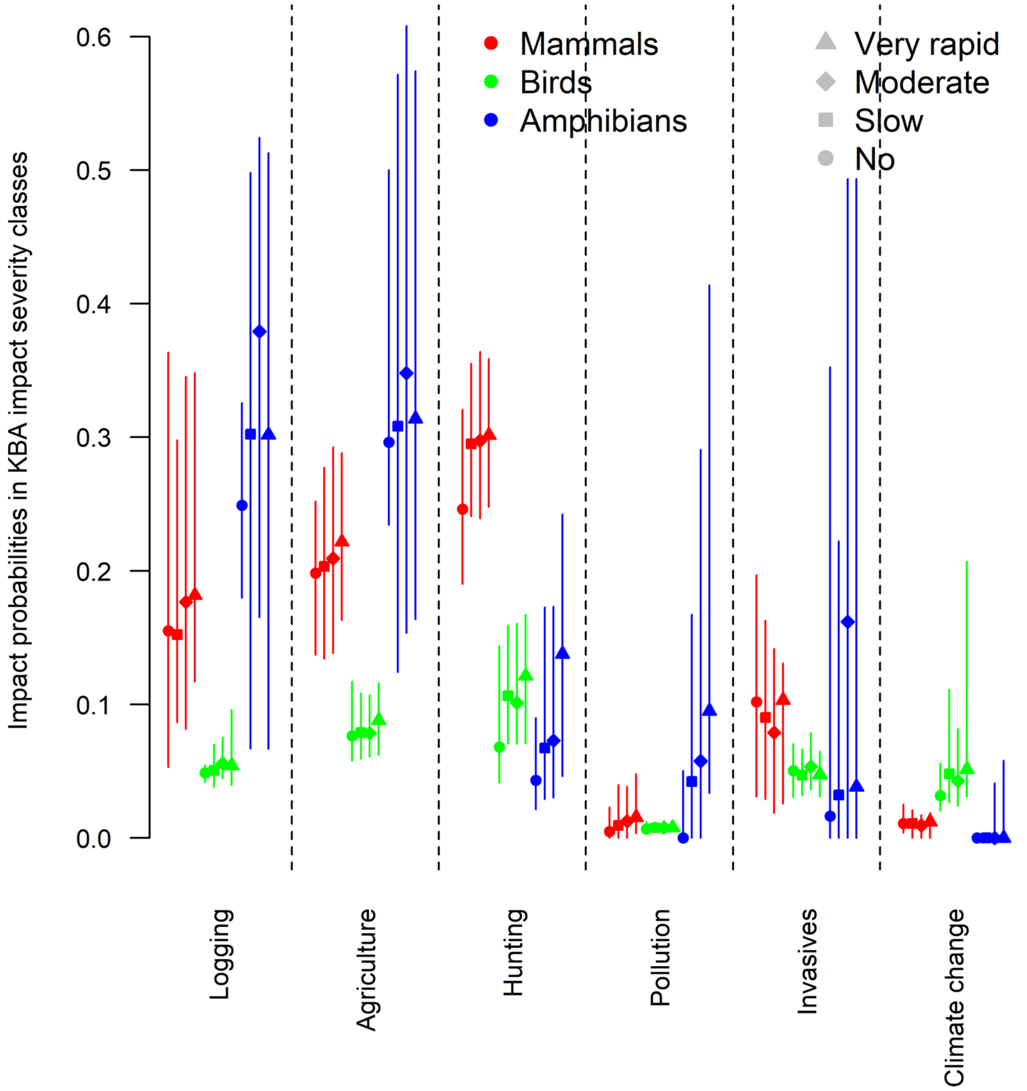

Supplementary Figure 9. Distributions (lines) and medians (points) for predicted impact probability across pixels intersecting with Key Biodiversity Areas (KBAs) grouped by the KBA impact severity class (No = "no or imperceptible deterioration", Slow = "slow but significant deterioration", Moderate = "moderate to rapid deterioration" and Very rapid = "very rapid to severe deterioration") and human activity class, for a binomial regression model weighted by the cube root of species range size.

Supplementary Figure 10.

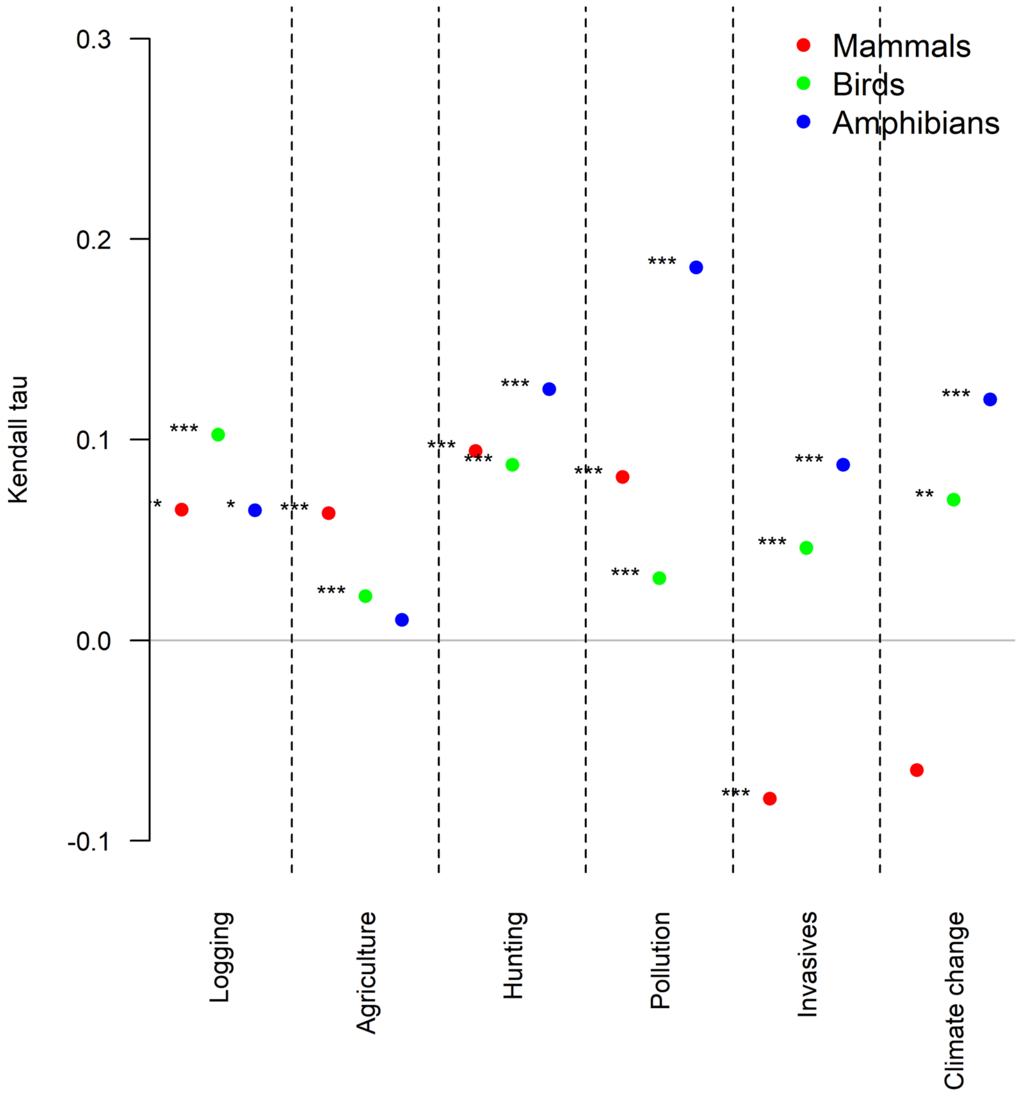

Supplementary Figure 10. Kendall's rank correlation coefficient, τ, for the predicted impact probability inside Key Biodiversity Areas (KBAs) using binomial regression weighted by the cube root of species range size against the rank of severity of impact in that KBA ("no or imperceptible deterioration" = 1, "slow but significant deterioration" = 2, "moderate to rapid deterioration" = 3, "very rapid to severe deterioration" = 4). Stars indicate levels of significance: * indicates $p < 0.1$, ** indicates $p < 0.05$ and *** indicates $p < 0.01$.

Supplementary Figure 11.

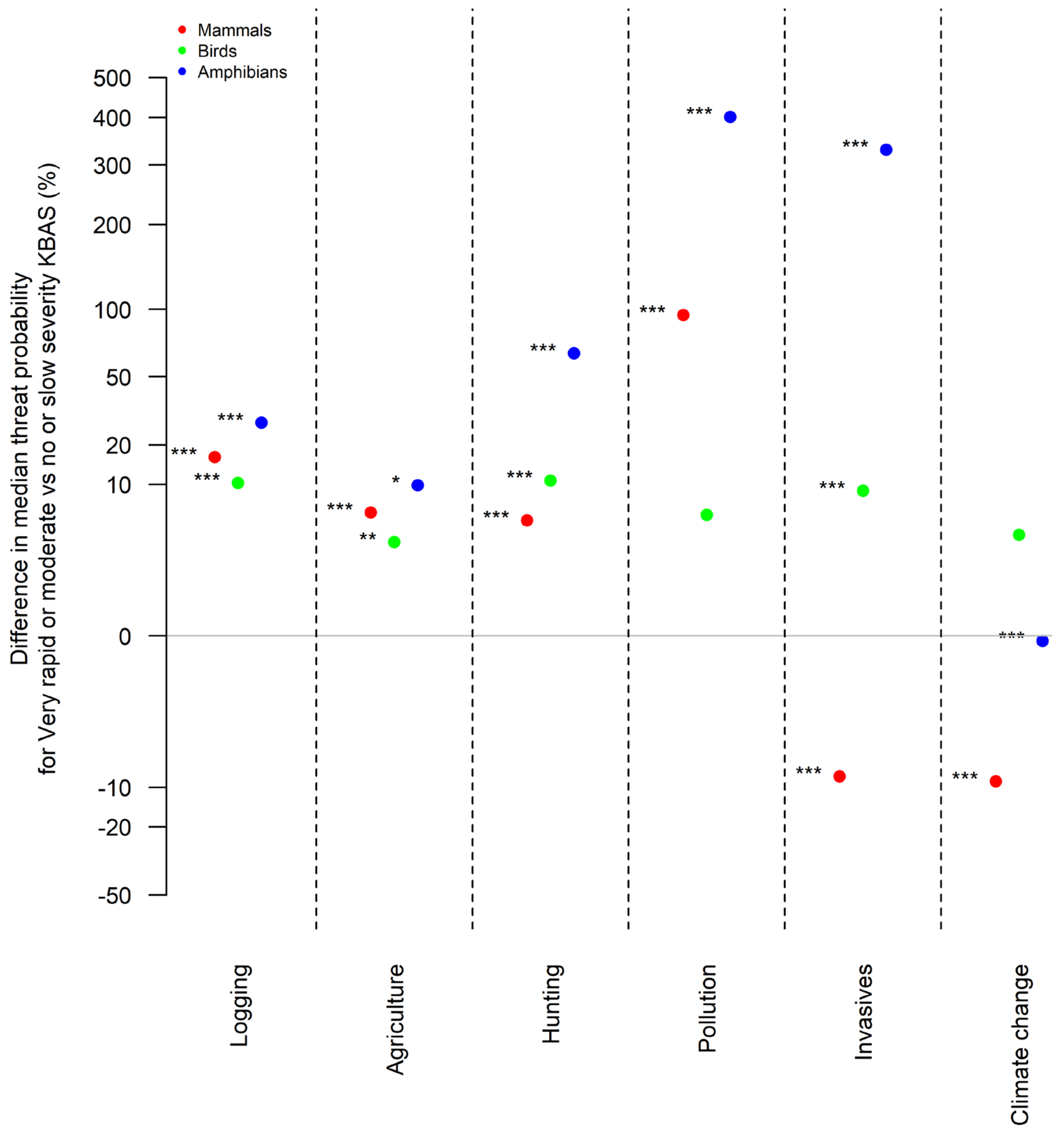

Supplementary Figure 11. The percentage difference in median predicted impact probability inside Key Biodiversity Areas (KBAs) assessed as having very rapid or moderate impact severity (very rapid = "very rapid to severe deterioration" and moderate = "moderate to rapid deterioration") compared with the value in KBAs classed as having no or low impact severity (no = "no or imperceptible deterioration" and slow = "slow but significant deterioration") for a binomial regression model weighted by the cube root of species range size. Values greater than a ratio of 0 (lying above the grey line) indicated that impact probability is predicted to be higher, on average, in KBAs that are classified as having high threat severity, compared to the predicted level of impact probability in KBAs classed as having low threat severity. Stars indicate levels of significance from Wilcox tests: * indicates $p < 0.1$, ** indicates $p < 0.05$ and *** indicates $p < 0.01$. Counts of KBAs used in the analysis were 72 and 88 for low and high severity threats respectively for logging; 360 and 201 for agriculture; 240 and 207 for hunting; 309 and 93 for pollution; 201 and 93 for invasives; and, 87 and 70 for climate change.

Supplementary Figure 12.

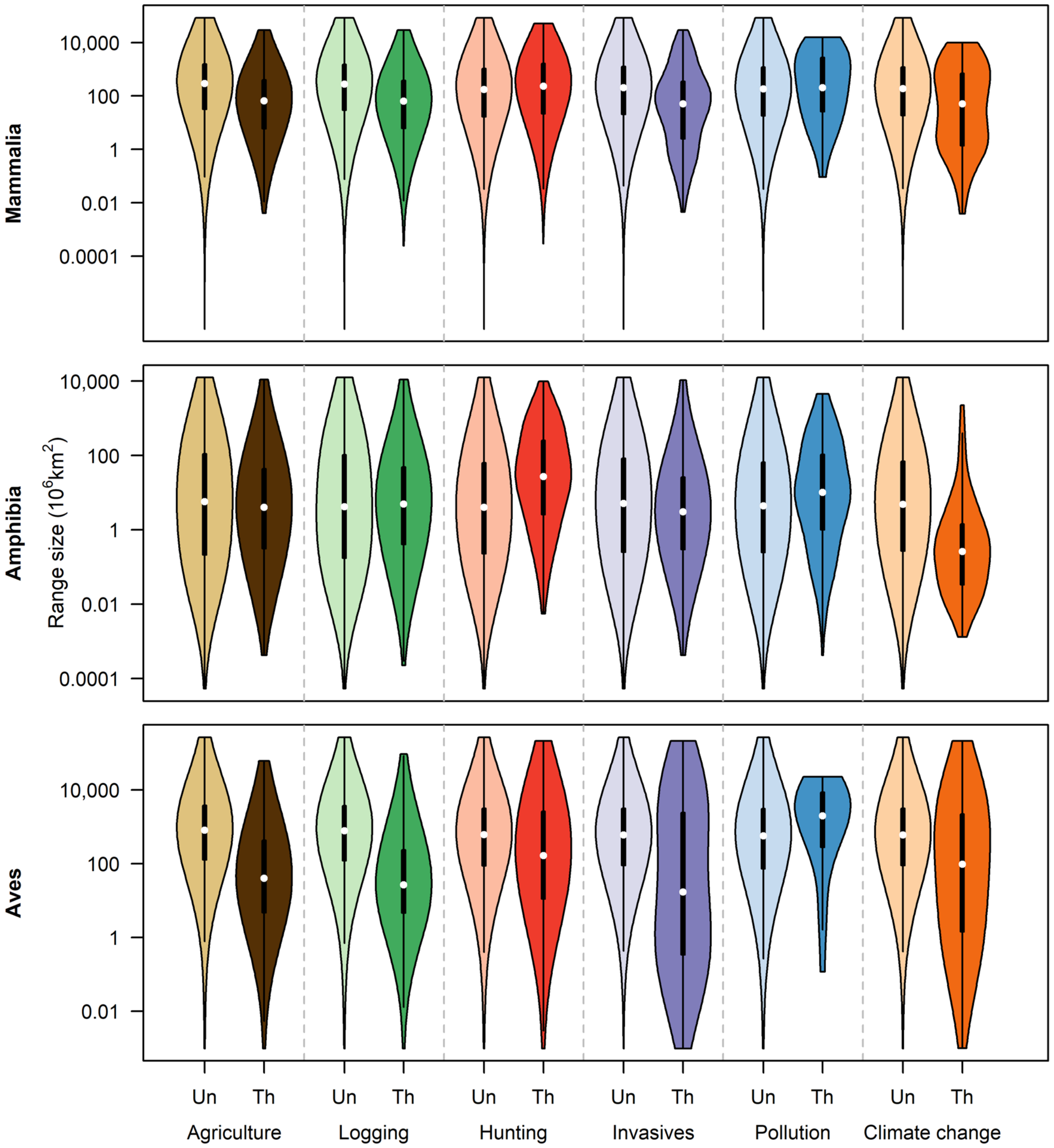

Supplementary Figure 12. The distributions of range sizes associated with species grouped according to whether they are assessed as being not impacted ('Un') or impacted ('Th') by principal threatening activities.

Supplementary Figure 13.

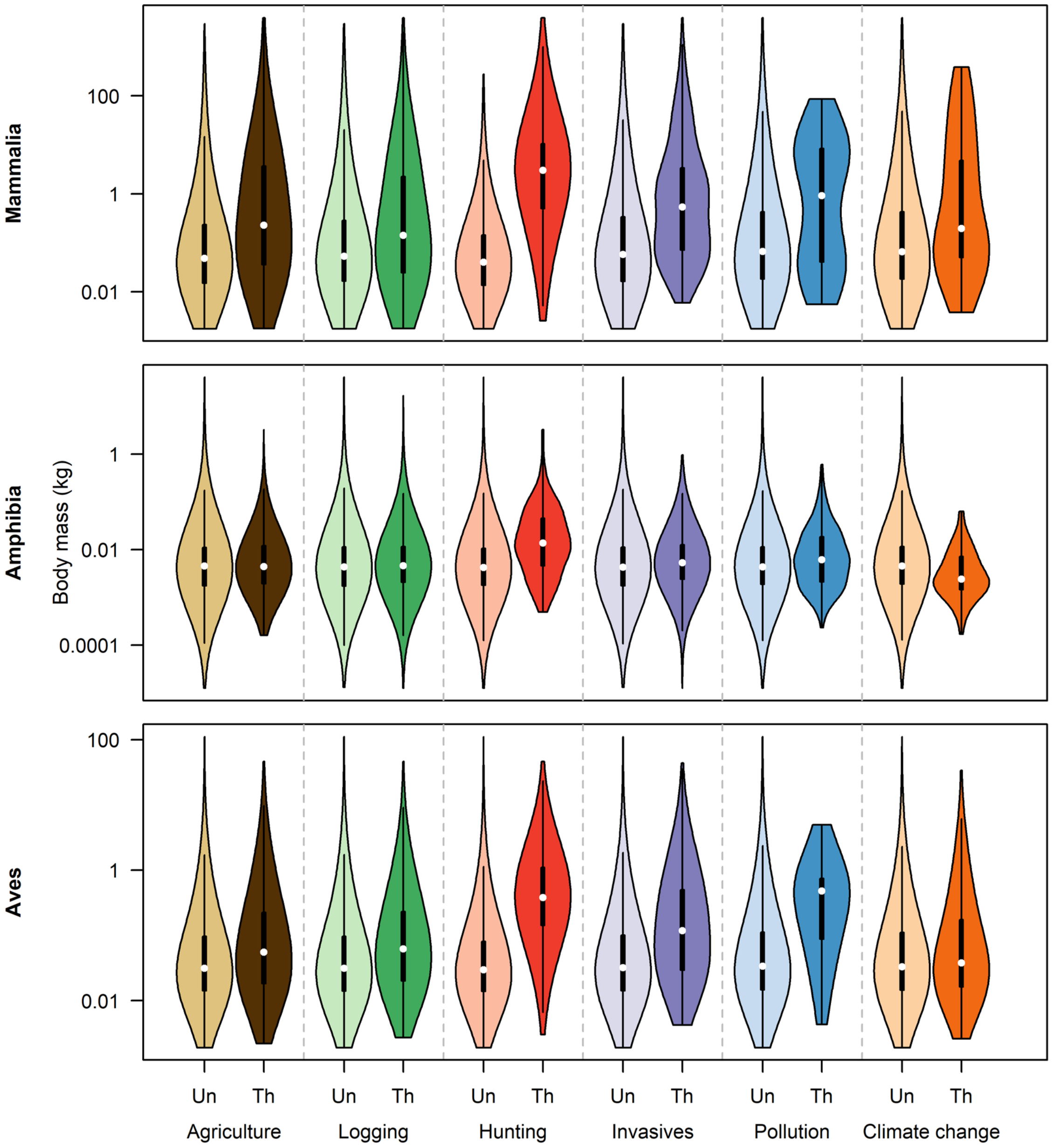

Supplementary Figure 13. The distributions of body sizes associated with species grouped according to whether they are assessed as being not impacted ('Un') or impacted ('Th') by principal threatening activities.

Supplementary Figure 14.

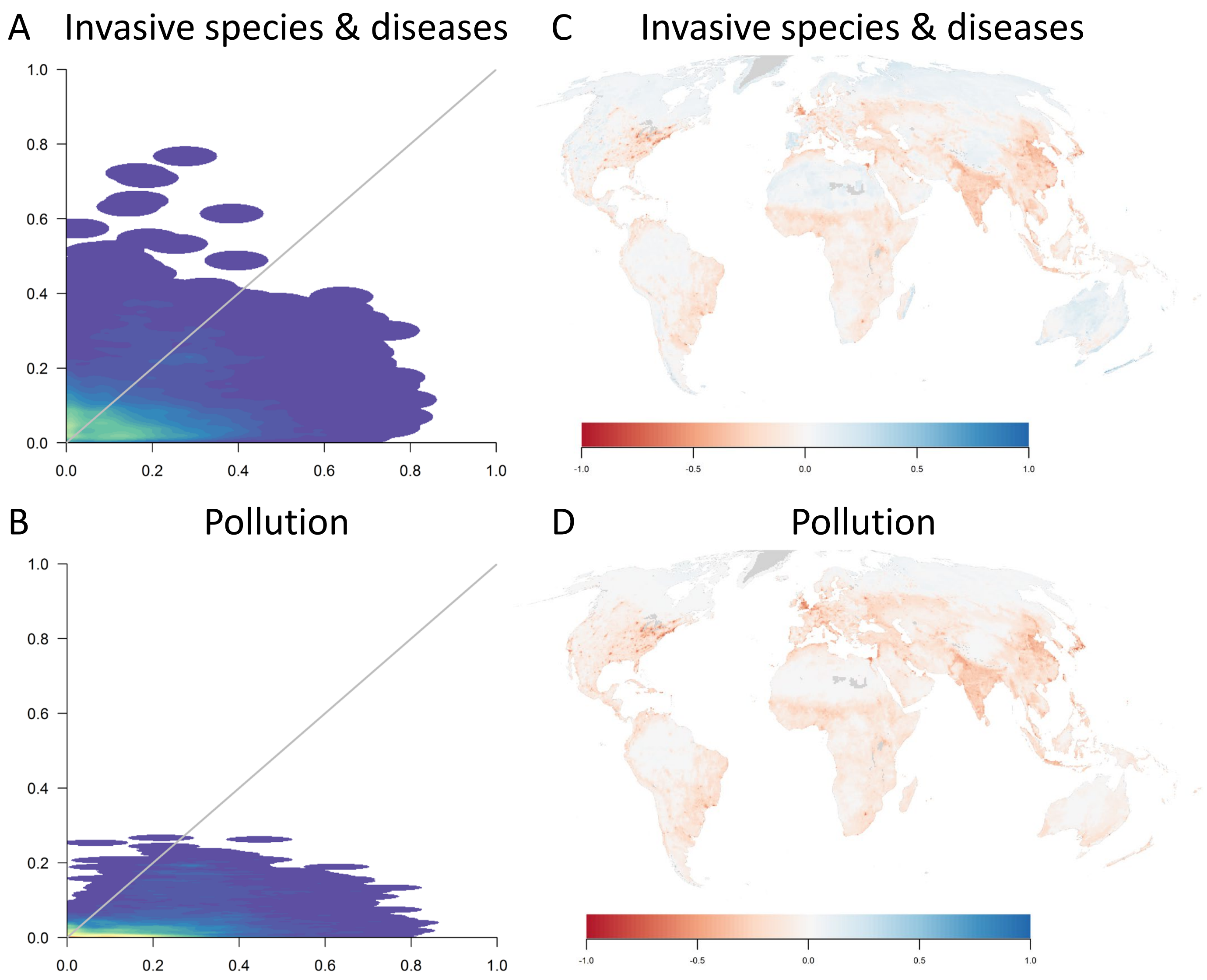

Supplementary Figure 14. Relationship between the Human Footprint and the probability of threats estimated from Red List data for amphibians, birds and mammals for invasive species & diseases (A) and for pollution (B). Grey lines indicate a 1:1 linear relationship. C and D show residuals from unity. Negative values (red colours) indicate where the Human Footprint might overestimate threat and, conversely, positive values (blue colours) indicate possible underestimation of threat.

Supplementary Table 1. List of models evaluated for threat probability mapping

**Description**	**Model structure**	**RMSE$_{med}$, r = 1×10^{-6}**	**RMSE$_{med}$, r = 1×10^{-4}**	**RMSE$_{med}$, r = 0.05**	**RMSE$_{med}$, r = 0.3**
1. Proportion of species present that are threatened by the activity	$P_{Th} \sim 1$	0.228	0.197	0.180	0.181
2. Binomial regression of probability of threat with species range size as a covariate	$P_{Th} \sim R$	0.229	0.192	0.196	0.184
3. Binomial regression of probability of threat with the natural logarithm of species range size as a covariate	$P_{Th} \sim ln(R)$	0.240	0.210	0.184	0.183
4. Binomial regression of probability of threat with square root species range size as a covariate	$P_{Th} \sim sqrt(R)$	0.233	0.198	0.186	0.184
5. Binomial regression of probability of threat with the inverse of range size as a weight	$P_{Th} \sim 1, weight = 1/R$	0.233	0.203	0.214	0.234
6. Binomial regression of probability of threat with the inverse of the natural logarithm of range size as a weight	$P_{Th} \sim 1, weight = 1/ln(R)$	0.225	0.193	0.179	0.180
7. Binomial regression of probability of threat with the inverse of cube root range size as a weight	$P_{Th} \sim 1, weight = 1/ R^{1/3}$	0.215	0.177	0.175	0.184
8. Binomial regression of probability of threat with the inverse of square root range size as a weight	$P_{Th} \sim 1, weight = 1/sqrt(R)$	0.210	0.173	0.174	0.190
9. Binomial regression of probability of threat with the inverse of 2.5- root range size as a weight	$P_{Th} \sim 1, weight = 1/ R^{10/25}$	0.213	0.175	0.174	0.185

Supplementary Table 2. List of models evaluated for threat probability mapping and their ranks (*Rk*) across permutations of sources of uncertainty associated with simulated threat data.

Map type	Uncertainty	$P_{Th} \sim 1$	$P_{Th} \sim R$	$P_{Th} \sim ln(R)$	$P_{Th} \sim sqrt(R)$	$P_{Th} \sim 1$, weight = 1/R	$P_{Th} \sim 1$, weight = 1/ln(R)	$P_{Th} \sim 1$, weight = $1/R^{1/3}$	$P_{Th} \sim 1$, weight = 1/sqrt(R)	$P_{Th} \sim 1$, weight = $1/R^{10/25}$
$r = 1\times10^{-6}$	$Rk_{Uncertain,0.25}$	7	6	9	8	1	5	4	2	3
	$Rk_{Uncertain,0.5}$	6	4	9	7	8	5	3	1	2
	$Rk_{Uncertain,0.75}$	5	6	8	7	9	3	1	4	2
	$Rk_{0.25}$	6	7	9	8	1	5	4	2	3
	$Rk_{0.5}$	6	7	9	8	4	5	3	1	2
	$Rk_{0.75}$	4	6	8	7	9	3	1	5	2
$r = 1\times10^{-4}$	$Rk_{Uncertain,0.25}$	8	5	9	7	1	6	4	2	3
	$Rk_{Uncertain,0.5}$	7	5	8	6	9	4	1	3	2
	$Rk_{Uncertain,0.75}$	5	6	8	7	9	4	3	1	2
	$Rk_{0.25}$	7	5	9	8	1	6	4	2	3
	$Rk_{0.5}$	7	3	8	5	9	6	1	4	2
	$Rk_{0.75}$	6	4	9	7	8	5	3	1	2
r = 0.05	$Rk_{Uncertain,0.25}$	7	5	9	8	1	6	4	2	3
	$Rk_{Uncertain,0.5}$	3	1	7	4	9	2	5	8	6
	$Rk_{Uncertain,0.75}$	5	8	7	6	9	4	2	3	1
	$Rk_{0.25}$	6	4	9	7	8	5	3	1	2
	$Rk_{0.5}$	5	1	8	6	9	4	2	7	3
	$Rk_{0.75}$	5	8	6	7	9	4	3	2	1
r = 0.3	$Rk_{Uncertain,0.25}$	6	4	8	7	9	5	3	1	2
	$Rk_{Uncertain,0.5}$	1	5	3	4	9	2	6	8	7
	$Rk_{Uncertain,0.75}$	5	1	7	4	9	3	2	8	6
	$Rk_{0.25}$	1	8	3	6	9	2	4	7	5
	$Rk_{0.5}$	1	5	3	6	9	2	4	8	7
	$Rk_{0.75}$	7	1	8	6	9	5	2	4	3
**Cumulative rank**		**126**	**115**	**181**	**156**	**168**	**101**	**72**	**87**	**74**

Supplementary Table 3. Summaries for models for allometric scaling between $\log_{10}$ transformed snout to vent length and $\log_{10}$ transformed bodymass for the amphibian orders, Anura, Caudata and Gymophiona.

Order	Intercept estimate	Slope estimate	P value	Adjusted r^2	Sample size, N
Anura	-3.29	2.47	< 1E-5	0.69	580
Caudata	-3.07	1.83	< 1E-5	0.62	113
Gymnophiona	-13.69	5.75	0.003	0.66	10

Supplementary Table 4. Coefficients of binomial regression models of assessed threat from each anthropogenic activity and the range size of species.

Taxa	Activity	Estimate	Std. Error	z-value	Pr(z)
Mammals	Agriculture	-1.21×10^{-4}	1.53×10^{-5}	-7.94	2.00×10^{-15}
Mammals	Logging	-1.53×10^{-4}	1.79×10^{-5}	-8.58	9.11×10^{-18}
Mammals	Hunting	5.08×10^{-5}	9.46×10^{-6}	5.37	8.02×10^{-8}
Mammals	Invasives	-2.39×10^{-5}	2.00×10^{-5}	-1.20	0.23
Mammals	Pollution	4.38×10^{-5}	2.35×10^{-5}	1.86	6.22×10^{-2}
Mammals	Climate change	-5.57×10^{-5}	5.40×10^{-5}	-1.03	0.30
Amphibians	Agriculture	-2.82×10^{-4}	3.74×10^{-5}	-7.55	4.25×10^{-14}
Amphibians	Logging	-2.46×10^{-4}	3.58×10^{-5}	-6.87	6.54×10^{-12}
Amphibians	Hunting	1.19×10^{-4}	3.99×10^{-5}	2.97	2.95×10^{-3}
Amphibians	Invasives	-3.66×10^{-4}	7.68×10^{-5}	-4.77E	1.85×10^{-6}
Amphibians	Pollution	-3.53×10^{-5}	7.12×10^{-5}	-0.50	0.62
Amphibians	Climate change	-1.86×10^{-3}	8.51×10^{-4}	-2.18	2.91×10^{-2}
Birds	Agriculture	-1.10×10^{-4}	1.06×10^{-5}	-10.4	3.61×10^{-25}
Birds	Logging	-1.50×10^{-4}	1.43×10^{-5}	-10.5	1.45×10^{-25}
Birds	Hunting	9.45×10^{-6}	2.53×10^{-6}	3.74	1.84×10^{-4}
Birds	Invasives	1.68×10^{-5}	2.60×10^{-6}	6.47	9.62×10^{-11}
Birds	Pollution	6.60×10^{-6}	1.26×10^{-5}	0.53	0.60
Birds	Climate change	7.44×10^{-6}	2.86×10^{-6}	2.61	9.12×10^{-3}

Supplementary Table 5. Coefficients of binomial regression models of assessed threat from each anthropogenic activity and the adult body mass of species.

Taxa	Activity	Estimate	Std. Error	z-value	Pr(z)
Mammals	Agriculture	2.74×10^{-3}	7.68×10^{-4}	3.57	3.53×10^{-4}
Mammals	Logging	8.32×10^{-4}	3.55×10^{-4}	2.34	1.91×10^{-2}
Mammals	Hunting	9.46×10^{-2}	7.20×10^{-3}	13.13	2.36×10^{-39}
Mammals	Invasives	1.16×10^{-3}	3.55×10^{-4}	3.27	1.08×10^{-3}
Mammals	Pollution	-2.16×10^{-5}	1.64×10^{-3}	-0.01	0.99
Mammals	Climate change	6.46×10^{-4}	5.28×10^{-4}	1.22	0.22
Amphibians	Agriculture	-1.12	5.08×10^{-1}	-2.21	2.69×10^{-2}
Amphibians	Logging	-8.45×10^{-2}	8.99×10^{-2}	-0.94	0.35
Amphibians	Hunting	3.44×10^{-2}	5.88×10^{-2}	0.58	0.56
Amphibians	Invasives	-9.13×10^{-1}	7.25×10^{-1}	-1.26	0.21
Amphibians	Pollution	-1.00×10^{-1}	3.35×10^{-1}	-0.30	0.77
Amphibians	Climate change	-2.70×10^{1}	13.9	-1.95	5.17×10^{-2}
Birds	Agriculture	6.88×10^{-2}	2.00×10^{-2}	3.44	5.92×10^{-4}
Birds	Logging	4.78×10^{-2}	1.79×10^{-2}	2.66	7.75×10^{-3}
Birds	Hunting	5.46×10^{-1}	3.75×10^{-2}	14.56	5.19×10^{-48}
Birds	Invasives	8.91×10^{-2}	2.13×10^{-2}	4.18	2.97×10^{-5}
Birds	Pollution	3.26×10^{-2}	3.39×10^{-2}	0.96	0.34
Birds	Climate change	2.51×10^{-2}	1.47×10^{-2}	1.72	8.63×10^{-2}

References

1 BirdLife International & NatureServe. Bird species distribution maps of the world. BirdLife International, Cambridge, United Kingdom and NatureServe, Arlington, United States. (2017).

2 IUCN. Red List of threatened species v 2017.3. (2017).

3 IUCN and The Conservation Measures Partnership. IUCN – CMP Threats Classification Scheme, version 3.2. 20 (The International Union for the Conservation of Nature and The Conservation Measures Partnership, Gland, Switzerland, 2019).

4 IUCN Standards and Petitions Committee. Guidelines for Using the IUCN Red List Categories and Criteria. Version 14. 113 (IUCN, Gland, Switzerland, 2019).

5 IUCN. Rules of Procedure for IUCN Red List Assessments 2017–2020. Version 3.0. 37 (IUCN, Gland, Switzerland, 2016).

6 Hansen, M. C. *et al.* High-Resolution Global Maps of 21st-Century Forest Cover Change. *Science* **342**, 850-853, doi:10.1126/science.1244693 (2013).

7 BirdLife International. (ed International Union for the Conservation of Nature the KBA Partnership: BirdLife International, American Bird Conservancy, Amphibian Survival Alliance, Conservation International, Critical Ecosystem Partnership Fund, Global Environment Facility, Global Wildlife Conservation, NatureServe, Rainforest Trust, Royal

Society for the Protection of Birds, Wildlife Conservation Society and World Wildlife Fund) (2019).
8 Joppa, L. N. *et al.* Filling in biodiversity threat gaps. *Science* **352**, 416-418, doi:10.1126/science.aaf3565 (2016).
9 IPBES. Summary for policymakers of the global assessment report on biodiversity and ecosystem services of the Intergovernmental Science-Policy Platform on Biodiversity and Ecosystem Services. 39 (Intergovernmental Science-Policy Platform on Biodiversity and Ecosystem Services, 2019).
10 Green, R. E., Cornell, S. J., Scharlemann, J. P. W. & Balmford, A. Farming and the Fate of Wild Nature. *Science* **307**, 550-555, doi:10.1126/science.1106049 (2005).
11 Salafsky, N. *et al.* A Standard Lexicon for Biodiversity Conservation: Unified Classifications of Threats and Actions. *Conservation Biology* **22**, 897-911, doi:10.1111/j.1523-1739.2008.00937.x (2008).
12 Olson, D. M. *et al.* Terrestrial Ecoregions of the World: A New Map of Life on Earth. *Bioscience* **51**, 933-938, doi:10.1641/0006-3568(2001)051[0933:teotwa]2.0.co;2 (2001).
13 Benítez-López, A., Santini, L., Schipper, A. M., Busana, M. & Huijbregts, M. A. J. Intact but empty forests? Patterns of hunting-induced mammal defaunation in the tropics. *PLOS Biology* **17**, e3000247, doi:10.1371/journal.pbio.3000247 (2019).
14 Redford, K. H. The Empty Forest. *Bioscience* **42**, 412-422 (1992).
15 Stokstad, E. The empty forest. *Science* **345**, 396-399, doi:10.1126/science.345.6195.396 (2014).
16 Wilkie, D. S., Bennett, E. L., Peres, C. A. & Cunningham, A. A. The empty forest revisited. *Annals of the New York Academy of Sciences* **1223**, 120-128, doi:10.1111/j.1749-6632.2010.05908.x (2011).
17 BirdLife International. Monitoring Important Bird Areas: a global framework version 1.2. (BirdLife International, Cambridge, UK, 2006).
18 Jetz, W., Carbone, C., Fulford, J. & Brown, J. H. The Scaling of Animal Space Use. *Science* **306**, 266-268, doi:10.1126/science.1102138 (2004).
19 Ofstad, E. G., Herfindal, I., Solberg, E. J. & Sæther, B.-E. Home ranges, habitat and body mass: simple correlates of home range size in ungulates. *Proceedings of the Royal Society B: Biological Sciences* **283**, 20161234, doi:doi:10.1098/rspb.2016.1234 (2016).
20 Barnes, M. *et al.* Wildlife population trends in protected areas predicted by national socio-economic metrics and body size. *Nature communications* **7**, 12747, doi:10.1038/ncomms12747 (2016).
21 Wilman, H. *et al.* EltonTraits 1.0: Species-level foraging attributes of the world's birds and mammals. *Ecology* **95**, 2027-2027, doi:10.1890/13-1917.1 (2014).
22 Oliveira, B. F., São-Pedro, V. A., Santos-Barrera, G., Penone, C. & Costa, G. C. AmphiBIO, a global database for amphibian ecological traits. *Scientific Data* **4**, 170123, doi:10.1038/sdata.2017.123 (2017).
23 Santini, L., Benítez-López, A., Ficetola, G. F. & Huijbregts, M. A. J. Length–mass allometries in amphibians. *Integrative Zoology* **13**, 36-45, doi:10.1111/1749-4877.12268 (2018).
24 Isaac, N. J. B. & Cowlishaw, G. How species respond to multiple extinction threats. *Proc Biol Sci* **271**, 1135-1141, doi:10.1098/rspb.2004.2724 (2004).
25 Fritz, S. A., P., B.-E. O. R. & Purvis, A. Geographical variation in predictors of mammalian extinction risk: big is bad, but only in the tropics. *Ecology Letters* **12**, 538-549 (2009).

26 Venter, O. *et al.* Sixteen years of change in the global terrestrial human footprint and implications for biodiversity conservation. *Nature communications* **7**, doi:10.1038/ncomms12558 (2016).

27 Venables, W. N. & Ripley, B. D. *Modern Applied Statistics with S. Fourth Edition.* . (Springer, 2002).
