# Supplementary material for: Using the IUCN Red List to map threats to terrestrial vertebrates at global scale
Source: Nat Ecol Evol. 2021 Aug 30;5(11):1510–9. doi: 10.1038/s41559-021-01542-9 (PMC8560638; doi:10.1038/s41559-021-01542-9)
Supplement: Supplementary file 2 — Reporting Summary [file 41559_2021_1542_MOESM2_ESM.pdf]

nature portfolio

Corresponding author(s): M Harfoot; J Geldmann

Last updated by author(s): Jul 7, 2021

Reporting Summary

Nature Portfolio wishes to improve the reproducibility of the work that we publish. This form provides structure for consistency and transparency in reporting. For further information on Nature Portfolio policies, see our Editorial Policies and the Editorial Policy Checklist.

Statistics

For all statistical analyses, confirm that the following items are present in the figure legend, table legend, main text, or Methods section.

n/a	Confirmed	
☐	☒	The exact sample size (n) for each experimental group/condition, given as a discrete number and unit of measurement
☒	☐	A statement on whether measurements were taken from distinct samples or whether the same sample was measured repeatedly
☐	☒	The statistical test(s) used AND whether they are one- or two-sided<br>*Only common tests should be described solely by name; describe more complex techniques in the Methods section.*
☐	☒	A description of all covariates tested
☐	☒	A description of any assumptions or corrections, such as tests of normality and adjustment for multiple comparisons
☐	☒	A full description of the statistical parameters including central tendency (e.g. means) or other basic estimates (e.g. regression coefficient) AND variation (e.g. standard deviation) or associated estimates of uncertainty (e.g. confidence intervals)
☐	☒	For null hypothesis testing, the test statistic (e.g. F, t, r) with confidence intervals, effect sizes, degrees of freedom and P value noted<br>*Give P values as exact values whenever suitable.*
☒	☐	For Bayesian analysis, information on the choice of priors and Markov chain Monte Carlo settings
☒	☐	For hierarchical and complex designs, identification of the appropriate level for tests and full reporting of outcomes
☐	☒	Estimates of effect sizes (e.g. Cohen's d, Pearson's r), indicating how they were calculated

Our web collection on statistics for biologists contains articles on many of the points above.

Software and code

Policy information about availability of computer code

Data collection	No software was used
Data analysis	R version 3.6.3 (x64) was used for data analysis and visualisations. Code is available from the github repository https://github.com/mikeharfoot/ThreatMapping

For manuscripts utilizing custom algorithms or software that are central to the research but not yet described in published literature, software must be made available to editors and reviewers. We strongly encourage code deposition in a community repository (e.g. GitHub). See the Nature Portfolio guidelines for submitting code & software for further information.

Data

Policy information about availability of data

All manuscripts must include a data availability statement. This statement should provide the following information, where applicable:

- Accession codes, unique identifiers, or web links for publicly available datasets
- A description of any restrictions on data availability
- For clinical datasets or third party data, please ensure that the statement adheres to our policy

Data on range maps are freely available at https://www.iucnredlist.org/resources/spatial-data-download and http://datazone.birdlife.org/species/requestdis. IUCN threat classification assessment data can be downloaded using the Red List API (http://apiv3.iucnredlist.org/api/v3/docs) or on request from IUCN’s Global Species Programme Red List Unit (redlist@iucn.org). Other data are freely available using citations in the manuscript.

Field-specific reporting

Please select the one below that is the best fit for your research. If you are not sure, read the appropriate sections before making your selection.

☐ Life sciences ☐ Behavioural & social sciences ☒ Ecological, evolutionary & environmental sciences

For a reference copy of the document with all sections, see nature.com/documents/nr-reporting-summary-flat.pdf

Ecological, evolutionary & environmental sciences study design

All studies must disclose on these points even when the disclosure is negative.

Study description	We use expert-derived information from the IUCN Red List on the range of species occurrence and threat classification assessment for 23,271 species, representing all terrestrial amphibians, birds and mammals, to estimate impact probability for six major threats to these groups: agriculture, hunting & trapping, logging, invasive species, pollution, and climate change. The study uses expert-derived spatial data and binary threat assessment data. Our study is spatially explicit, using a 50km x 50km global grid. The modelling approach was developed using simulated data then evaluated using datasets most closely related to spatial threat distribution: global deforestation data (Hansen et al., Science, 2013) and threat assessments for Key Biodiversity Areas. Models of the impact probability were parameterised independently for each pixel and were only considered valid where there were more than 10 species present within the grid cell.
Research sample	Our sample was all terrestrial amphibians, birds and mammals as assessed by the IUCN Red List.
Sampling strategy	We chose to use the comprehensively assessed taxonomic groups within the IUCN Red List, in order to avoid spatial bias in modelling that might result from sampling bias towards locations where taxa are more likely to be assessed. These groups have also been assessed against the Red List Threat Classification scheme.
Data collection	Data was obtained from the IUCN Red List as described in the Data availability statement above
Timing and spatial scale	The Red List Assessment process if an assessment of the threat status of contemporary organisms and is continually being updated. We used the Red List version 2017-3. The data is global in scale.
Data exclusions	When fitting models for each of the groups (amphibians, birds or mammals) data was excluded where it occurred in spatial pixels for which fewer than 10 species from that taxonomic group were estimated to occur in that cell. This threshold was used as a conservative estimate of the data needed to constrain our simple models and to avoid spurious results.
Reproducibility	We did not conduce experiments, however we have ensured that the data used in our analysis and the code used for the analysis is freely available.
Randomization	Organisms were grouped according to their taxonomy reflecting qualitatively different ways in which they may be be impacted by human activities.
Blinding	Blinding was not relevant for our study because we were not testing for differential effects between groups

Did the study involve field work? ☐ Yes ☒ No

Reporting for specific materials, systems and methods

We require information from authors about some types of materials, experimental systems and methods used in many studies. Here, indicate whether each material, system or method listed is relevant to your study. If you are not sure if a list item applies to your research, read the appropriate section before selecting a response.

Materials & experimental systems

n/a	Involved in the study	
☒	☐	Antibodies
☒	☐	Eukaryotic cell lines
☒	☐	Palaeontology and archaeology
☒	☐	Animals and other organisms
☒	☐	Human research participants
☒	☐	Clinical data
☒	☐	Dual use research of concern

Methods

n/a	Involved in the study	
☒	☐	ChIP-seq
☒	☐	Flow cytometry
☒	☐	MRI-based neuroimaging
