# Supplementary material for: Using the IUCN Red List to map threats to terrestrial vertebrates at global scale
Source: Nat Ecol Evol. 2021 Aug 30;5(11):1510–9. doi: 10.1038/s41559-021-01542-9 (PMC8560638; doi:10.1038/s41559-021-01542-9)
Supplement: Supplementary file 3 — Peer Review Information [file 41559_2021_1542_MOESM3_ESM.pdf]

Peer Review Information

Journal: Nature Ecology & Evolution
Manuscript Title: Using the IUCN Red List to map threats to terrestrial vertebrates at global scale
Corresponding author name(s): Michael B. J. Harfoot, Jonas Geldmann

Editorial Notes:

**Redactions – transferred manuscripts (mention of previous referee reports from elsewhere)**	This manuscript has been previously reviewed at another journal. This document only contains reviewer comments, rebuttal and decision letters for versions considered at Nature Ecology & Evolution. Mentions of prior referee reports have been redacted
**Redactions – transferred manuscripts (mention of the other journal)**	This manuscript has been previously reviewed at another journal. This document only contains reviewer comments, rebuttal and decision letters for versions considered at Nature Ecology & Evolution. Mentions of the other journal have been redacted.

Reviewer Comments & Decisions:

Decision Letter, initial version:

28th March 2021

*Please ensure you delete the link to your author homepage in this e-mail if you wish to forward it to your co-authors.

Dear Dr Geldmann,

Thank you for transferring your manuscript entitled "Towards a global map of threats to species" to Nature Ecology & Evolution. As I communicated by email to your co-author Mike Harfoot last week, we are pleased to offer publication in NEE, subject to some further requested revisions ***[REDACTED]***. We

do not expect to engage those reviewers again, nor to recruit additional reviewers, but will need to see these responses and your revised manuscript before we can reach a final decision regarding publication.

We are committed to providing a fair and constructive peer-review and editorial process. Do not hesitate to contact us if you have queries about the below requests. ***[REDACTED]***.

Requested revisions:

1. The paper will need to be reformatted to NEE style; we don't require reformatting to consider transferred papers, but we do like authors to undertake some reformatting prior to publication. This is mostly because our papers can be somewhat longer than allowed for ***[REDACTED]*** papers, and we usually find that this additional length is helpful to add discussion around points raised during review.

Our Articles can be up to around 3500 words, or a little longer if necessary. This is the length for the main text (not including abstract, references or methods). Please structure the main text as an Introduction section followed by a Results & Discussion section (retaining the subheadings is fine).

The abstract can be 150-200 words. If you feel it would help to slightly lengthen your abstract, please do so within this limit.

We aim for Methods sections to be up to around 3000 words, but they are not word limited and in general we prefer methods to be fully in the main text rather than split between main and supplementary methods, if this is achievable within the 3000 word ballpark. In this case, I suspect all of the Methods could go in the main paper or perhaps only the KBA analysis should be retained in the Supplementary Information.

We also allow up to 10 Extended Data figures that are viewed in-line in the main text of the paper online. Please consider which of your current SI figures should be converted to Extended Data and which retained in the supplement.

2. We hope that our longer format will allow some additional space for upfront addressing of the two main remaining concerns of the reviewers: the use of expert-derived data, and the current limitations of the maps presented.

To address the first point, we would like to see more of your response to the concerns around potential bias from expert-derived data placed in the main text rather than in the Supplementary Information. For example, from line 196, I think it would be helpful to expand somewhat on why you are confident in the use of Red List expert-derived information as being appropriate for the maps you produce. There was a lot of information in the 'response to reviewers' letter that helped me to understand both the reviewer concerns and your confidence. I'm not suggesting that all of this needs to go into the main text, but I think some additional discussion would be genuinely helpful to readers.

To address the second point, I would like to see some acknowledgement that the presented maps are not suitable for local conservation decision making earlier in the manuscript – perhaps at the start of the section 'Priorities for threat mitigation', or elsewhere if you feel there is another spot that is more appropriate. I also felt similarly to Reviewer #4 that some of the wording in your section on "Improving Future Threat Mapping" gave the impression "of an added ambition (a predictive accuracy

at the local scale)”. Please could you further clarify this section to address whether this approach would ever be applicable for informing local-level conservation planning – I think it is fine to state that this would never be the goal of this approach, but that it is complementary to local-level approaches. But I do think it is ambiguous at the moment.

3. I am also still somewhat concerned by the use of the phrase ‘high predictive accuracy’ at line 305 and, to a lesser extent, at line 73. Would ‘concordance’ be an appropriate word to use instead of ‘predictive accuracy’? If so, I feel that might help to avoid potential misinterpretation of threat-prediction capability.

4. Please could you consider a more direct, informative title. Perhaps: “Global terrestrial threat maps derived from expert-informed Red List data” – or something in that vein.

When you do resubmit, it would be very helpful if you could please provide the document as a word doc as well as a pdf, so that I can look at lengths.

When revising your manuscript:

* Include a response document detailing, point-by-point, how you addressed the above requests. If no action was taken to address a point, you must provide a compelling argument.

* If you have not done so already please begin to revise your manuscript so that it conforms to our Article format instructions at http://www.nature.com/natecolevol/info/final-submission. Refer also to any guidelines provided in this letter.

* Include a revised version of any required reporting checklist.

Please use the link below to submit your revised manuscript and related files:

[REDACTED]

Note: This URL links to your confidential home page and associated information about manuscripts you may have submitted, or that you are reviewing for us. If you wish to forward this email to co-authors, please delete the link to your homepage.

We hope to receive your revised manuscript within four to eight weeks. If you cannot send it within this time, please let us know. We will be happy to consider your revision so long as nothing similar has been accepted for publication at Nature Ecology & Evolution or published elsewhere.

Nature Ecology & Evolution is committed to improving transparency in authorship. As part of our efforts in this direction, we are now requesting that all authors identified as ‘corresponding author’ on published papers create and link their Open Researcher and Contributor Identifier (ORCID) with their account on the Manuscript Tracking System (MTS), prior to acceptance. ORCID helps the scientific community achieve unambiguous attribution of all scholarly contributions. You can create and link your ORCID from the home page of the MTS by clicking on ‘Modify my Springer Nature account’. For more information please visit please visit www.springernature.com/orcid.

We look forward to seeing the revised manuscript and thank you for the opportunity to consider your work.

[REDACTED]

Decision Letter, first revision:

16th May 2021

Dear Jonas,

Thank you for submitting your revised manuscript "Using the IUCN Red List to map threats to terrestrial vertebrates at global scale" (NATECOLEVOL-210313262A). We appreciate the revisions you made - thank you for making them so clear in the letter and identifying them in the text. We are now happy in principle to publish it in Nature Ecology & Evolution, pending some minor revisions to comply with our editorial and formatting guidelines.

If the current version of your manuscript is in a PDF format only, please email us a copy of the file in an editable format (Microsoft Word or LaTex)-- we can not proceed with PDFs at this stage.

The editorial assistant and I will now perform detailed checks on your paper and will send you a checklist detailing our editorial and formatting requirements in about a week. Please do not upload the final materials and make any revisions until you receive this additional information from us.

[REDACTED]

Our ref: NATECOLEVOL-210313262A

21st May 2021

Dear Dr. Geldmann,

Thank you for your patience as we've prepared the guidelines for final submission of your Nature Ecology & Evolution manuscript, "Using the IUCN Red List to map threats to terrestrial vertebrates at global scale" (NATECOLEVOL-210313262A). Please carefully follow the step-by-step instructions provided in the attached file, and add a response in each row of the table to indicate the changes that you have made. Please also check and comment on any additional marked-up edits we have proposed within the text. Ensuring that each point is addressed will help to ensure that your revised manuscript can be swiftly handed over to our production team.

**We would like to start working on your revised paper, with all of the requested files and forms, as

soon as possible (preferably within two weeks). Please get in contact with us immediately if you anticipate it taking more than two weeks to submit these revised files.**

When you upload your final materials, please include a point-by-point response to any remaining reviewer comments.

If you have not done so already, please alert us to any related manuscripts from your group that are under consideration or in press at other journals, or are being written up for submission to other journals (see: https://www.nature.com/nature-research/editorial-policies/plagiarism#policy-on-duplicate-publication for details).

In recognition of the time and expertise our reviewers provide to Nature Ecology & Evolution's editorial process, we would like to formally acknowledge their contribution to the external peer review of your manuscript entitled "Using the IUCN Red List to map threats to terrestrial vertebrates at global scale". For those reviewers who give their assent, we will be publishing their names alongside the published article.

Nature Ecology & Evolution offers a Transparent Peer Review option for new original research manuscripts submitted after December 1st, 2019. As part of this initiative, we encourage our authors to support increased transparency into the peer review process by agreeing to have the reviewer comments, author rebuttal letters, and editorial decision letters published as a Supplementary item. When you submit your final files please clearly state in your cover letter whether or not you would like to participate in this initiative. Please note that failure to state your preference will result in delays in accepting your manuscript for publication.

Cover suggestions

As you prepare your final files we encourage you to consider whether you have any images or illustrations that may be appropriate for use on the cover of Nature Ecology & Evolution.

Covers should be both aesthetically appealing and scientifically relevant, and should be supplied at the best quality available. Due to the prominence of these images, we do not generally select images featuring faces, children, text, graphs, schematic drawings, or collages on our covers.

We accept TIFF, JPEG, PNG or PSD file formats (a layered PSD file would be ideal), and the image should be at least 300ppi resolution (preferably 600-1200 ppi), in CMYK colour mode.

If your image is selected, we may also use it on the journal website as a banner image, and may need to make artistic alterations to fit our journal style.

Please submit your suggestions, clearly labeled, along with your final files. We'll be in touch if more information is needed.

Nature Ecology & Evolution has now transitioned to a unified Rights Collection system which will allow our Author Services team to quickly and easily collect the rights and permissions required to publish your work. Approximately 10 days after your paper is formally accepted, you will receive an email in providing you with a link to complete the grant of rights. If your paper is eligible for Open Access, our

Author Services team will also be in touch regarding any additional information that may be required to arrange payment for your article.

Please note that <i>Nature Ecology & Evolution</i> is a Transformative Journal (TJ). Authors may publish their research with us through the traditional subscription access route or make their paper immediately open access through payment of an article-processing charge (APC). Authors will not be required to make a final decision about access to their article until it has been accepted. Find out more about Transformative Journals

<B>Authors may need to take specific actions to achieve compliance with funder and institutional open access mandates. For submissions from January 2021, if your research is supported by a funder that requires immediate open access (e.g. according to Plan S principles) then you should select the gold OA route, and we will direct you to the compliant route where possible. For authors selecting the subscription publication route our standard licensing terms will need to be accepted, including our self-archiving policies. Those standard licensing terms will supersede any other terms that the author or any third party may assert apply to any version of the manuscript.

Please note that you will not receive your proofs until the publishing agreement has been received through our system.

For information regarding our different publishing models please see our Transformative Journals page. If you have any questions about costs, Open Access requirements, or our legal forms, please contact ASJournals@springernature.com.

Please use the following link for uploading these materials:
[REDACTED]

If you have any further questions, please feel free to contact me.

[REDACTED]

Final Decision Letter:

15th July 2021

Dear Jonas,

We are pleased to inform you that your Article entitled "Using the IUCN Red List to map threats to

terrestrial vertebrates at global scale", has now been formally accepted for publication in Nature Ecology & Evolution.

Before your manuscript is typeset, our subeditor will edit the text to ensure it conforms to house style. Once your manuscript is typeset you will receive a link to your electronic proof via email, with a request to make any corrections within 48 hours. If you have queries at any point during the production process then please contact the production team at rjsproduction@springernature.com. Once your paper has been scheduled for online publication, the Nature press office will be in touch to confirm the details.

Acceptance of your manuscript is conditional on all authors' agreement with our publication policies (see www.nature.com/authors/policies/index.html). In particular your manuscript must not be published elsewhere and there must be no announcement of the work to any media outlet until the publication date (the day on which it is uploaded onto our web site).

Please note that <i>Nature Ecology & Evolution</i> is a Transformative Journal (TJ). Authors may publish their research with us through the traditional subscription access route or make their paper immediately open access through payment of an article-processing charge (APC). Authors will not be required to make a final decision about access to their article until it has been accepted. Find out more about Transformative Journals

<B>Authors may need to take specific actions to achieve compliance with funder and institutional open access mandates. For submissions from January 2021, if your research is supported by a funder that requires immediate open access (e.g. according to Plan S principles) then you should select the gold OA route, and we will direct you to the compliant route where possible. For authors selecting the subscription publication route our standard licensing terms will need to be accepted, including our self-archiving policies. Those standard licensing terms will supersede any other terms that the author or any third party may assert apply to any version of the manuscript.

In approximately 10 business days you will receive an email with a link to choose the appropriate publishing options for your paper and our Author Services team will be in touch regarding any additional information that may be required.

You will not receive your proofs until the publishing agreement has been received through our system.

If you have any questions about our publishing options, costs, Open Access requirements, or our legal forms, please contact ASJournals@springernature.com

An online order form for reprints of your paper is available at https://www.nature.com/reprints/author-reprints.html. All co-authors, authors' institutions and authors' funding agencies can order reprints using the form appropriate to their geographical region.

We welcome the submission of potential cover material (including a short caption of around 40 words) related to your manuscript; suggestions should be sent to Nature Ecology & Evolution as electronic files (the image should be 300 dpi at 210 x 297 mm in either TIFF or JPEG format). Please note that such pictures should be selected more for their aesthetic appeal than for their scientific content, and that colour images work better than black and white or grayscale images. Please do not try to design a cover with the Nature Ecology & Evolution logo etc., and please do not submit composites of images related to your work. I am sure you will understand that we cannot make any promise as to whether any of your suggestions might be selected for the cover of the journal.

You can now use a single sign-on for all your accounts, view the status of all your manuscript submissions and reviews, access usage statistics for your published articles and download a record of your refereeing activity for the Nature journals.

To assist our authors in disseminating their research to the broader community, our SharedIt initiative provides you with a unique shareable link that will allow anyone (with or without a subscription) to read the published article. Recipients of the link with a subscription will also be able to download and print the PDF.

You can generate the link yourself when you receive your article DOI by entering it here: <a href="http://authors.springernature.com/share">http://authors.springernature.com/share<a>.

Thank you again for choosing Nature Ecology & Evolution to publish your work, and for your patience throughout the process. It's been a pleasure working with you and Mike on it and I look forward to seeing it published soon.

[REDACTED]

P.S. Click on the following link if you would like to recommend Nature Ecology & Evolution to your librarian http://www.nature.com/subscriptions/recommend.html#forms

** Visit the Springer Nature Editorial and Publishing website at www.springernature.com/editorial-and-publishing-jobs for more information about our career opportunities. If you have any questions please click here.**
